# Mobile Technology Interventions for Asthma Self-Management: Systematic Review and Meta-Analysis

**DOI:** 10.2196/mhealth.7168

**Published:** 2017-05-02

**Authors:** Lisa Miller, Benjamin Schüz, Julia Walters, E Haydn Walters

**Affiliations:** ^1^ School of Medicine, Psychology University of Tasmania Hobart Australia; ^2^ University of Tasmania Hobart Australia

**Keywords:** asthma, mhealth, medication adherence, patient monitoring, behavior and behavior mechanisms, meta-analysis

## Abstract

**Background:**

Mobile technology interventions (MTI) are becoming increasingly popular in the management of chronic health behaviors. Most MTI allow individuals to monitor medication use, record symptoms, or store and activate disease-management action plans. Therefore, MTI may have the potential to improve low adherence to medication and action plans for individuals with asthma, which is associated with poor clinical outcomes.

**Objective:**

A systematic review and meta-analysis were conducted to evaluate the efficacy of MTI on clinical outcomes as well as adherence in individuals with asthma. As the use of evidence-based behavior change techniques (BCT) has been shown to improve intervention effects, we also conducted exploratory analyses to determine the role of BCT and engagement with MTI as moderators of MTI efficacy.

**Methods:**

We searched electronic databases for randomized controlled trials up until June 2016. Random effect models were used to assess the effect of MTI on clinical outcomes as well as adherence to preventer medication or symptom monitoring. Mixed effects models assessed whether the features of the MTI (ie, use of BCT) and how often a person engaged with MTI moderated the effects of MTI.

**Results:**

The literature search located 11 studies meeting the inclusion criteria, with 9 providing satisfactory data for meta-analysis. Compared with standard treatment, MTI had moderate to large effect sizes (Hedges *g*) on medication adherence and clinical outcomes. MTI had no additional effects on adherence or clinical outcomes when compared with paper-based monitoring. No moderator effects were found, and the number of studies was small. A narrative review of the two studies, which are not included in the meta-analysis, found similar results.

**Conclusions:**

This review indicated the efficacy of MTI for self-management in individuals with asthma and also indicated that MTI appears to be as efficacious as paper-based monitoring. This review also suggested a need for robust studies to examine the effects of BCT use and engagement on MTI efficacy to inform the evidence base for MTI in individuals with asthma.

## Introduction

### Background

Asthma is a chronic inflammatory disease of the airways that is characterized physiologically by excessive variation in airflow and manifests symptomatically as repeated episodes of coughing, wheezing, shortness of breath, and chest tightness [1]. Asthma affects 2.5 million Australians (11%) across the lifespan [2].

In terms of treatment recommendations, the National Asthma Council Australia and Global Strategy for Asthma Management and Prevention have recommended the use of personalized action plans and daily preventer medications to manage the illness [1,3]. Action plans are associated with improved clinical outcomes through incorporation of appropriate education, self-monitoring of symptoms and medication use, and review of symptoms by the individual between physician visits [4]. In more severe patients, daily preventer treatment is associated with a reduced risk of death, as well as asthma exacerbations, thus requiring hospitalization or oral steroid with their related side effects [5,6].

Despite current guidelines recommending action plans and daily preventer medication, patients’ actual adherence to either treatment remains low. In 2015, only 28.10% (702,500/ 2,500,000) of Australians with asthma had an action plan [2]. Similarly, medication adherence is also suboptimal, with only 59.63% (1601/2685) of those with asthma reported using preventer medication at least once within a 12-month period, and only 33.89% (910/2685) reporting daily use [6]. Objective data on the dispensing of prescriptions for asthma preventer medication indicate that less than 18.00% (253,123/1,406,240) of those with asthma use preventer medication daily [7]. A longitudinal study following middle-aged adults with asthma over 12 months found that 73.8% (259/351) used inadequate preventer medication [8]. This is concerning, as research suggests that appropriate use of preventer medication could protect against progressive decline in lung function, which is associated with increasing asthma severity [8].

Lower adherence is associated with individuals who perceive their asthma to be an acute condition with few adverse personal consequences and thus has a lower perceived necessity for the preventer treatment [9]. The Common Sense Model of Illness [10] provides a theoretical explanation for asthma nonadherence. Information provided to the individual should inform perceptions of the causes, consequences, controllability, identity, and timeline of asthma, which drive coping responses around adherence, management, and so on. This in turn should as a consequence have positive effects on clinical outcomes, that is, exacerbation rates, hospitalizations, and so on [10].

### Mobile Technology Interventions

Mobile technology interventions (MTI) can provide an external source of self-management support, allow for accurate real-time symptom and medication monitoring, and use built-in reminders to adhere to treatment, as well as can store and activate action plans. Studies suggest that over 80% of people with asthma are willing to use MTI, and quantitative studies suggest that it is an acceptable medium for assisting asthma self-management [11,12]. This might be facilitated by MTI providing a feeling of support that positively influences individuals’ ability to cope with their asthma [13], for example, by identifying asthma-related stressors [11]. It is also suggested that MTI may improve the quality of care and asthma-management skills as well as allow for greater understanding of client attitudes, interpretation, and misconceptions of their asthma management [14]. Further, MTI have shown positive short-term behavioral outcomes for self-management medication in diabetes and for antiretroviral therapy [15]. However, physicians have highlighted major concerns to MTI use including increased time and resource demands, accuracy of information, liability, and patient confidentiality [14].

Research on behavioral interventions suggests that emphasizing specific behavior change techniques (BCT) in MTI allows clearly defined objectives for intervention development and therefore improved replication [16]. A recent review of mobile intervention applications for medication adherence found that action plans, prompt or cues, self-monitoring, and feedback on behavior were the most commonly used BCT, but at the same time that most recent mobile applications made limited or no use of BCT [17]. Using BCT in MTI has several advantages: As observable working components of MTI, BCT allow for replication and creation of a sustainable evidence base—basically, it becomes easier to compare MTI efficacy based on their contents and develop more effective MTI on this evidence base. Further, the theoretical underpinning of BCT allows understanding and identifying the mechanisms of change in adherence behavior and informs future improved MTI development [16]. BCT can therefore increase the likelihood of MTI efficacy to improve medication adherence [17], in particular, if they are being deployed within a well-functioning health care provider-patient relationship [11], and the BCT align with the therapeutic approach to self-management. Currently, it is unclear to what degree MTI for asthma management apply evidence-based BCT or provide evidence-based content.

Behaviour change techniques, including action plans, self-monitoring, and feedback on behavior, may be unsuccessful unless the individual engages with the MTI. Engagement with the MTI is in itself a quantifiable measure, ie, how often the individual sends or responds to the MTI [18], and indeed the efficacy of the MTI is likely to be dependent on the active engagement of the individual [19]. However, the reporting of such engagement with MTI has been poor, and engagement appears to be widely variable [18].

### MTI in Asthma: The Present Review

There have been two reviews of MTI in asthma self-management. Belisario and colleagues [20] conducted a systematic review and meta-analysis of two MTI of asthma self-management and found insufficient evidence to draw a conclusion. Tran and colleagues [21] conducted a systematic review of 6 MTI of asthma self-management and found MTI was associated with greater medication adherence compared with standard treatment, but this did not translate to improvements in quality of life, symptom control, or lung function. These studies highlighted the need to identify studies that included evidence-based techniques of MTI, which successfully instigated changes and sustainability of self-management behavior.

### Aims of This Review

This study therefore aimed to investigate the efficacy of MTI for adherence (medication and self-monitoring) and clinical health outcomes (lung function, quality of life, asthma control, and unscheduled visits) in individuals with asthma. In addition, we conducted explanatory analyses to answer the following important theoretical and applied research questions: (1) Is MTI more efficacious than standard treatment or paper-based monitoring? (2) What behaviour change techniques are effective via mobile technology interventionsI? (3) Does engagement with the MTI enhance outcomes?

## Methods

### Literature Search, Inclusion Criteria, and Study Selection

This review adheres to the Preferred Reporting Items for Systematic Reviews and Meta-Analyses (PRISMA, [22]). A systematic literature review was conducted using Medical Literature Analysis and Retrieval System Online (MEDLINE), PsychNet, Scopus, and Web of Science to collect published studies as well as ProQuest Dissertations and Theses Global and ClinicalTrials.gov for relevant trials or unpublished studies. In addition, the reference lists of included studies and applicable systematic reviews were hand searched to identify additional studies. Titles, abstracts, and keywords were used to identify relevant studies (refer to Multimedia Appendix 1 for the full search strategy).

**Figure 1:**
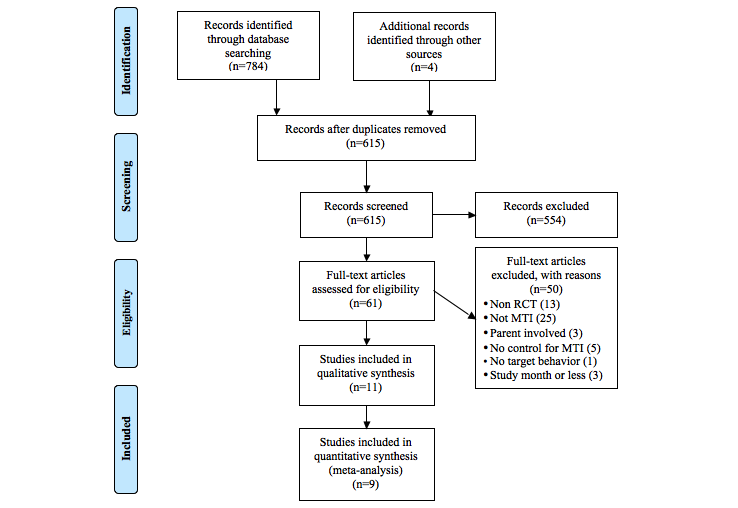
Flowchart of the study selection process for the systematic review and meta-analysis.

We included any randomized controlled trial using an MTI where the primary target of the MTI was the individual with asthma. Therefore, trials with children were excluded if the parent was responsible for asthma management. MTI were included if they used any mobile device that was currently available (ie, smart phone, tablet, or mobile phone) and any platform of delivery that incorporated at least one target behavior (ie, text message, application, music file). Papers not written in English were excluded. Screening individuals with asthma can result in improved asthma control over the proceeding week [23]. Therefore, to ensure that any changes in outcomes were due to the MTI, only the papers that exceeded the study duration of 1 month were included. The search was completed on the June 30, 2016, with the identification of 61 potential studies. Full texts of all eligible studies were retrieved and assessed against the inclusion criteria by LM and reviewed through discussion with BS. There were 11 studies included in the systematic review, with 9 included in the meta‐analysis, on the basis of sufficient data. A flowchart of the study selection process can be found in Figure 1. The studies excluded from the full-text review and their reasons for omission can be found in Multimedia Appendix 2.

### Study Characteristics

For each study, we extracted publication characteristics (eg, year of publication), the study design (eg, type of intervention, MTI characteristics), the sample (eg, female percentage, mean age, asthma severity), and the outcome variables (eg, adherence, symptom monitoring, lung function, quality of life) at pre- and postintervention or at change between pre- and postintervention time points. Data from the end point were taken for those studies reporting data over multiple times. A complete list of the extracted characteristics from all the identified studies included in the meta-analysis and the narrative review can be found in Multimedia Appendix 3.

### Classification of BCT

None of the included studies specifically mentioned any behaviour change technique. We used the behavior change technology taxonomy [16] to code the features of the MTI. This taxonomy identifies 93 distinct and evidenced-based techniques within 16 categories of behavior-change interventions. The features of any paper-based monitoring and standard treatment groups were also classified according to the taxonomy for each of the included studies.

### Risk of Bias Assessment

The quality of the included manuscripts was assessed according to the Cochrane Risk of Bias Guidelines [24]. The Cochrane Risk of Bias dimensions included random sequence generation, allocation concealment, blinding of outcome assessment, incomplete outcome data, selective reporting, and other bias [24]. The option of “blinding participants and personnel” was excluded because participants could not be blinded as to whether they did or did not receive the MTI. LM assessed the risk of bias in the included studies, which was reviewed through discussion with BS. Each domain was judged as “low,” “high,” or “unclear.”

### Primary and Secondary Outcomes

The primary outcomes were clinical health markers (ie, lung function, quality of life, asthma control, and unscheduled visits to the general physician or the emergency department) as well as frequency of self-monitoring and medication behaviors (adherence). Secondary outcomes were the type and number of behaviour change technique used and the frequency of mobile technology intervention use (engagement) *.*

### Meta-Analytic Strategy

The most frequently reported effects were postintervention unstandardized means and standard deviations, or change from, and therefore Hedges *g* was the effect measure used for the meta‐analysis. If available, change data were used in preference to postintervention data, as change scores provide a more powerful estimate of the intervention effect [24]. Bias-corrected Hedges *g* was reported to account for the positively biased estimate of an effect size when sample sizes are small, which was common in the included studies [25].

The meta-analytic procedure was conducted using the *R* “metafor” package [26] applying a random effects model on pooled effect sizes of primary and secondary outcomes. The assumption of the random effects model is that variability between effect sizes is due to real between-study differences and allows for generalizability of findings beyond the included studies [27]. It was hypothesized that studies in the meta-analysis would provide multiple effect sizes because different outcome variables were being assessed. The magnitude of the effect was interpreted as small (0.20), moderate (0.50), and large (0.80) [25].

Heterogeneity of effect sizes was measured using *Q* and *I*^2^statistics. Caution should be taken when interpreting these statistics as they are often imprecisely estimated in the presence of a small number of studies, as in this meta-analysis [24]. Funnel plots were visually examined to assess for publication bias. No tests of funnel plot asymmetry were conducted due to the small power of the test in a small sample of studies [24].

Mixed-effects meta-analysis was used to perform the categorical and continuous moderation analyses. Due to overlap in BCT between MTI and paper-based monitoring, only studies comparing MTI with standard treatment were included in the BCT moderation analyses. BCT were aggregated to 4 categorical variables (self-monitoring with feedback, self-monitoring only, prompting only, other) and were entered as categorical moderators. All relevant behavior change technique taxonomies (BCTT), with the exception of “Instruction on how to perform the behavior” (BCTT 4.1) and “Information about health consequences” (BCTT 5.1), contributed to the number of BCTT in the MTI group. Behavior change technique taxonomies 4.1 and 5.1 were excluded as they were also commonly used in the standard treatment group.

A categorical moderation analysis was used to assess the within- and between-group variability of effect sizes using a dummy-coded categorical factor to indicate the moderators. Categorical moderation is indicated by a significant between-group statistic (*QM*).

The numbers of BCT used in MTI and engagement with MTI were entered as continuous moderators for the effect size estimates, and the standardized regression coefficient of the moderator was examined for statistical significance. In addition, the *R*^2^statistic indicates how much of the variation between studies can be explained by the model containing the moderator, relative to the total variation.

## Results

### Study Characteristics

Table 1 shows the population characteristics of the included studies. The mean sample size of 11 included studies was 103, ranging from 16 to 288. The mean study duration was 17 weeks, ranging from 12 to 24 weeks. The age of participants ranged from 10 years to about 65 years, with a mean age of 34 years, and the percentage of female participants was 61.1% (583/954). Asthma severity varied from mild to severe persistent asthma, but the method used to assess this was reported only in 5 studies [28-32].

The effect sizes for MTI were computed from comparisons of MTI with paper-based and standard treatment. Three studies compared MTI with paper-based [29,31,32], one with paper-based as well as standard treatment [30], and the remaining studies compared MTI with standard treatment.

Four studies used MTI with a mobile app platform (MTI-App) and the remaining 7 used MTI with a short message service platform (MTI-SMS). Interactivity, that is, how often the person sent or received content from the MTI, ranged from daily to weekly, with daily being the most common (55%, 6/11) followed by twice daily (36%, 4/11). Seven of the studies used tailoring in the form of feedback based on individuals’ symptoms [28-34]. One study sent tailored SMS based on faulty illness perceptions [35]. Four studies explicitly stated the application of behavior change theories when designing their MTI [32,35-37]. These included the Health Belief Model [38], Illness Perceptions for Adherence [39], and Monitoring in Chronic Disease [40]. Seven studies did not report a theoretical model for their MTI [28-31,33,34,41].

**Table 1:** Study population characteristics of included studies.

Lead author	Year	Country	N (Duration in weeks)	Mean age in years (range)	Female %	Asthma severity
Ostojic [31]	2005	Croatia	16 (16)	25 (18+)	44 (7/16)	Moderate (?^a^)
Liu [29]	2007	Taiwan	120 (24)	52 (18+)	51 (45/89)	Moderate to severe (?)
Prabhakaran [33]	2010	Singapore	120 (12)	55 (21+)	59.2 (71/120)	Poorly controlled (94%)
Strandbygaard [41]	2010	Denmark	26 (12)	32 (18-45)	46 (12/26)	Moderate to severe (69%)
Lv [30]	2012	China	150 (12)	38 (18-65)	42 (30/71)	Moderate to severe (73%)
Petrie [35]	2012	UK	147 (18)	? (16-45)	68.0 (100/147)	Nonadherence (100%)
Ryan [32]	2012	UK	288 (24)	49 (12+)	62.5 (180/288)	Poorly controlled (100%)
Yun [37]	2012	USA	30 (15)	14 (10-16)	47 (7/15)	Moderate to severe (?)
Yun [36]	2013	USA	30 (16)	13 (10-16)	57 (12/21)	Moderate to severe (?)
Cingi [28]	2015	Turkey	136 (12)	33 (25-41)	53 (47/89)	Mild to severe (?)
Zairina [34]	2016	Australia	72 (24)	31 (18+)	100 (72/72)	Moderate to severe (58%)

^a^Value could not be identified in the study.

### Classification of BCT

There were 10 distinct behaviour change techniques identified within the included studies across 8 categories (Tables 2 and 3) “Instruction on how to monitor symptoms and medication” as well as “Information on health consequences” appeared to represent the standard education provided to asthma participants as part of their standard treatment and was used across all groups. All MTI-App used self-monitoring of symptoms or medication, compared with 44% (4/9) of MTI-SMS. Prompt or cues were used in 78% (7/9) of MTI-SMS compared with 25% (1/4) of MTI-App. Action planning was a lot more common in MTI-App (75%, 3/4) than MTI-SMS (11%, 1/9). Paper-based monitoring utilized similar BCT as MTI-App but did not provide feedback or social support from an external source. “Feedback” was categorized as individuals receiving feedback based on their symptoms or medication being monitored. “Social support” (practical) differed from feedback, as the participant could send a message to their physician or the investigators for responses to questions they had.

**Table 2:** Classification of behavior change technique taxonomy for each included study.

Lead author	Year	MTI^a^Platform	MTI	Comparator Type
Ostojic [31]	2005	MTI-SMS^b^	2.3; 2.6; 2.7; 3.2; 4.1; 5.1	Paper-based: 2.3; 4.1; 5.1
Liu [29]	2007	MTI-App^c^	1.4; 2.3; 2.6; 4.1; 5.1	Paper-based: 1.4; 2.3; 2.6; 4.1; 5.1
Prabhakaran [33]	2010	MTI-SMS	2.3; 2.7; 3.2; 4.1; 7.1	Standard treatment: 4.1
Strandbygaard [41]	2010	MTI-SMS	4.1; 5.1; 7.1	Standard treatment: 4.1; 5.1
Lv [30]	2012	MTI-SMS	1.4; 3.2; 4.1; 5.1; 7.1	Paper-based: 1.4; 2.3; 4.1; 5.1 Standard treatment: 4.1; 5.1
Petrie [35]	2012	MTI-SMS	4.2; 5.1	Standard treatment: ?
Ryan [32]	2012	MTI-App	1.4; 2.3; 2.7; 3.2; 4.1; 5.1; 6.1	Paper-based: 1.4; 2.3; 4.1; 6.1
Yun [37]	2012	MTI-SMS	2.3; 2.7; 5.1; 7.1	MTI: 2.3; 7.1 Standard treatment: ?
Yun [36]	2013	MTI-SMS	2.3; 2.7; 5.1; 7.1	MTI: 5.1; 7.1 Standard treatment: ?
Cingi [28]	2015	MTI-App	2.3; 3.2; 4.1; 7.1	Standard treatment: 4.1
Zairina [34]	2016	MTI-App	1.4; 2.3; 2.7; 3.2; 5.1	Standard treatment: 5.1

^a^MTI: Mobile technology interventions.

^b^MTI-SMS: Mobile technology interventions with short message service platform.

^c^MTI-App: Mobile technology interventions with mobile app platform.

**Table 3:** Behavior change technique taxonomy used by study group.

Behavior change technique taxonomy	MTI-App^a^ n (%) (N=4)	MTI-SMS^b^ n (%) (N=9)	MTI^c^-All n (%) (N=13)	Paper-based n (%) (N=4)	Standard treatment n (%) (N=8)
1.4 Action planning	3 (75%)	1 (11%)	4 (31%)	3 (75%)	0 (0%)
2.3 Self-monitoring of behavior	4 (100%)	4 (44%)	8 (62%)	4 (100%)	0 (0%)
2.6 Biofeedback	1 (25%)	1 (11%)	2 (15%)	1 (25%)	0 (0%)
2.7 Feedback on outcomes of behavior	2 (50%)	4 (44%)	6 (46%)	0 (0%)	0 (0%)
3.2 Social support (practical)	3 (75%)	3 (33%)	5 (46%)	0 (0%)	0 (0%)
4.1 Instruction on how to perform the behavior	3 (75%)	4 (44%)	7 (54%)	4 (100%)	4 (50%)
4.2 Information about antecedents	0 (0%)	1 (11%)	1 (8%)	0 (0%)	0 (0%)
5.1 Information about health consequences	3 (75%)	8 (89%)	11 (85%)	3 (75%)	3 (38%)
6.1 Demonstration of the behavior	1 (25%)	0 (0%)	1 (8%)	1 (25%)	0 (0%)
7.1 Prompts or cues	1 (25%)	7 (78%)	8 (62%)	0 (0%)	0 (0%)

^a^MTI-App: Mobile technology interventions with mobile app platform.

^b^MTI-SMS: Mobile technology interventions with short message service platform.

^c^MTI: Mobile technology interventions.

### Risk of Bias Assessment

**Figure 2:**
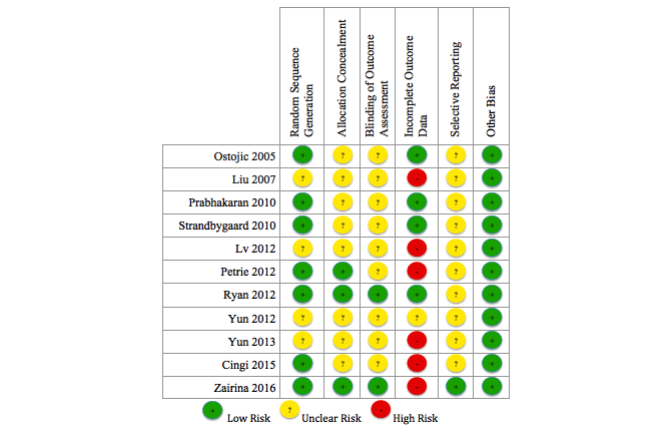
Cochrane risk of bias assessment: risk of bias dimension for each included study.

**Figure 3:**
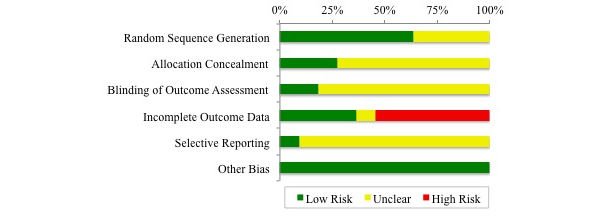
Cochrane risk of bias assessment: summary of each risk of bias item.

The results of the Cochrane Risk of Bias assessment are shown in Figures 2 and 3, with complete details presented in Multimedia Appendix 4.

Seven studies reported using appropriate random sequence generation methods (computer-generated random allocation, random order on presentation, or drawn from envelope); 4 studies did not specify the method of randomization [29,30,36,37]. Three studies reported concealment of allocation [32,34,35]. The remaining 8 studies were unclear as to whether allocation was concealed. Only 2 studies addressed blinding of participants and outcome assessors [32,34]. Although not stated, we assume that the remaining 9 studies did not carry out blinding of participants or outcome assessors.

Six of the studies had high risk of incomplete outcome data, due to varying rates of dropout or missing data between the groups [28,30,34,36], or because those who dropped out had higher medication adherence [35] or dropped out due to difficulty with MTI [29]. In one study, all participants completed the study [31], and the remaining 4 studies had low and similar dropout rates between the groups. One study provided a study protocol, which inferred low risk of selective reporting [34]. The remaining studies were unclear on potential selective reporting, but relevant variables appeared to be reported. No other potential sources of bias were identified. Overall the risk profile was quite reasonable.

### Clinical Outcomes

The studies reported adherence to medication as well as symptoms or diary entries over a time period between 12 and 24 weeks. Adherence was assessed either via prescription data or self-report throughout the intervention or at the end of the study. Four studies reported percentage adherence to preventer medication at end of study [30,32,35] or change over the study period [41]. The remaining two provided average inhaled cortico-steroid dosage at the end of study [29,31] *.* Two studies reported percentage adherence at end of study to symptom monitoring and action plan [29] and peak expiratory flow rate monitoring [31].

Five studies provided data on lung function in the form of mean Forced Expiratory Volume in 1 second predicted at end of the study [29,32] or change over the study period [30,34,41]. Four studies provided data on change in Quality of Life (QoL) over the study period, using the full or mini version of the Asthma-Specific Quality of Life Questionnaire [30,32,34,41]. One study provided mean scores, at the end of the study period only, using the Short-Form-12 Questionnaire—Physical Component Score [29]. Four studies provided data on change in asthma control over the study period, using the Asthma Control Questionnaire [32,34,41] and the Perceived Control of Asthma Questionnaire [30]. Three studies provided the percentage of individuals with well-controlled asthma at end of the study [28,33,34], while 6 studies reported the percentage of unscheduled visits at end of the study [28-33].

Five studies provided the mean percentage of engagement, which ranged from 72% to 99% [29,31,33,36,37]. Three studies did not provided data on engagement with the MTI [32,34] or only median data were available [28]. A further two studies [30,41] did not have adequate engagement data as the MTI incorporated an SMS prompt that did not require engagement with the system.

#### MTI Effectiveness by Adherence and Health Outcome

Table 4 shows the effect of MTI on adherence based on the random effects model. Tests of heterogeneity suggested low variation among the true effects between the remaining studies. However, the width of the confidence intervals suggests an imprecise estimate of heterogeneity and caution should be taken when interpreting these results.

**Figure 4:**
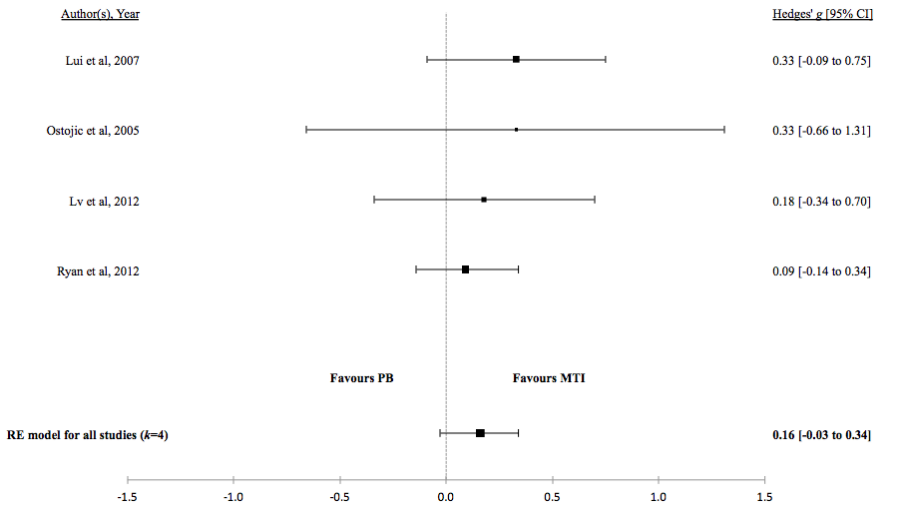
Forest plot of the standardized mean difference in medication adherence between MTI and Paper-based group (PB). MTI: mobile technology intervention.

**Figure 5:**
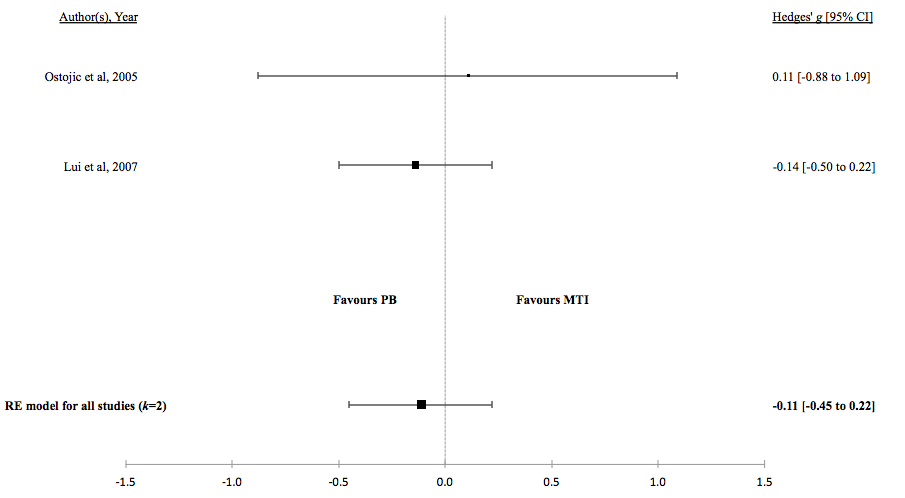
Forest plot of the standardized mean difference in symptom or diary adherence between MTI and paper-based group (PB). MTI: mobile technology intervention.

**Figure 6:**
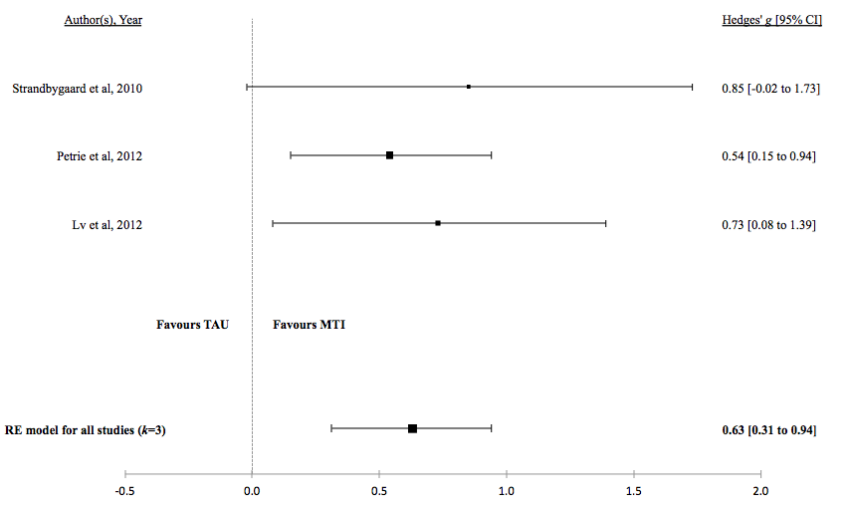
Forest plot of the standardized mean difference in medication adherence between MTI and standard treatment group. MTI: mobile technology intervention.

There was no evidence for a difference in the standardized mean medication adherence or symptom-monitoring adherence in studies comparing MTI with paper-based (Figures 4 and 5, respectively). Individuals using MTI had a significantly higher standardized mean medication adherence compared with standard treatment (Figure 6), suggesting a moderate positive effect.

Table 5 shows the effect of MTI on clinical outcomes based on the random effects model. Test of heterogeneity suggested high variation in asthma control between MTI and standard treatment studies as well as in unscheduled visits between MTI and paper-based studies. There was moderate to low variation among the true effects between the remaining studies, but the width of the confidence intervals suggests an imprecise estimate of heterogeneity and caution should be taken when interpreting these results.

**Figure 7:**
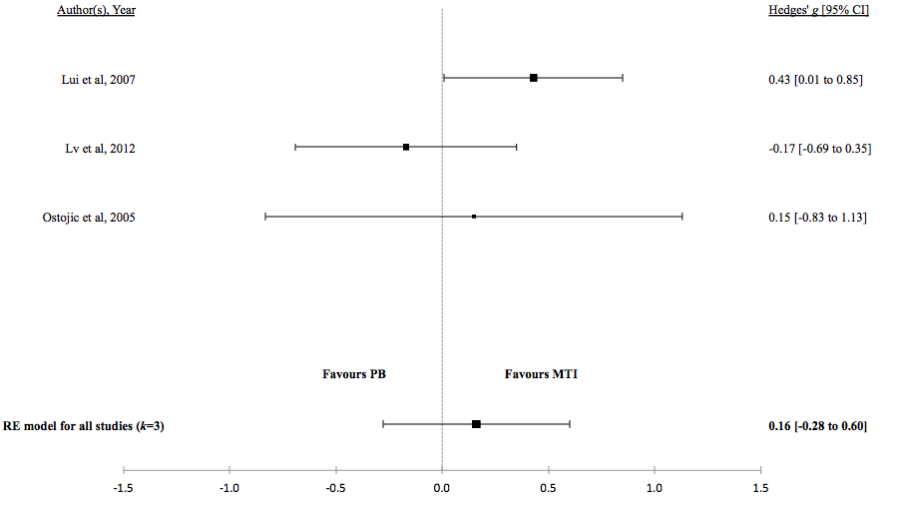
Forest plot of the standardized mean difference in lung function between MTI and paper-based group. MTI: mobile technology intervention.

**Figure 8:**
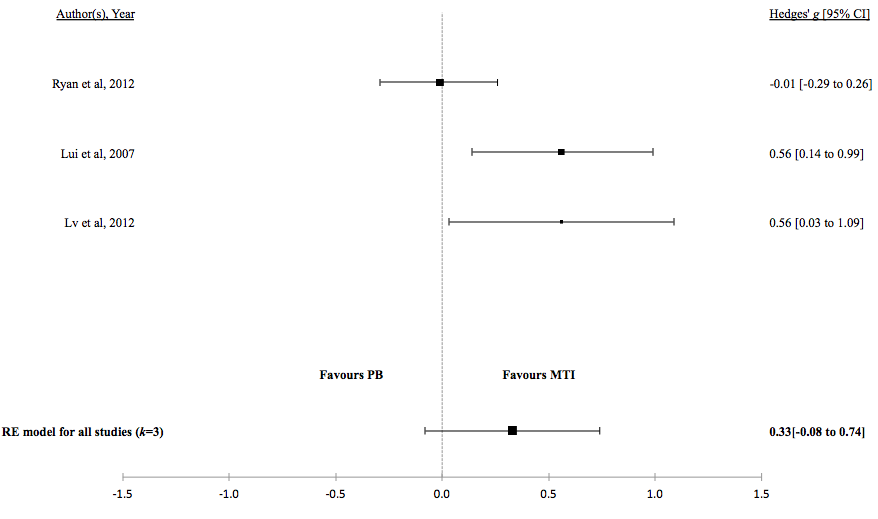
Forest plot of the standardized mean difference in quality of life between MTI and paper-based group (PB). MTI: mobile technology intervention.

**Figure 9:**
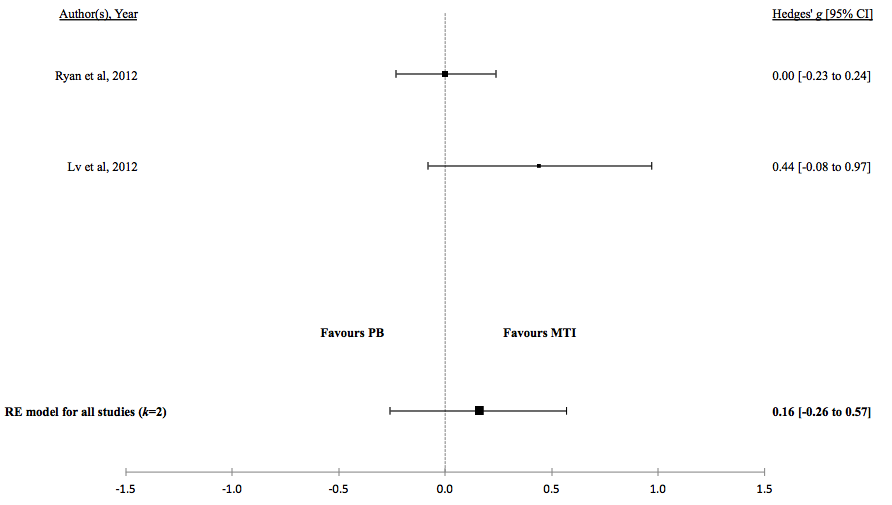
Forest plot of the standardized mean difference in asthma control between MTI and paper-based group (PB). MTI: mobile technology intervention.

**Figure 10:**
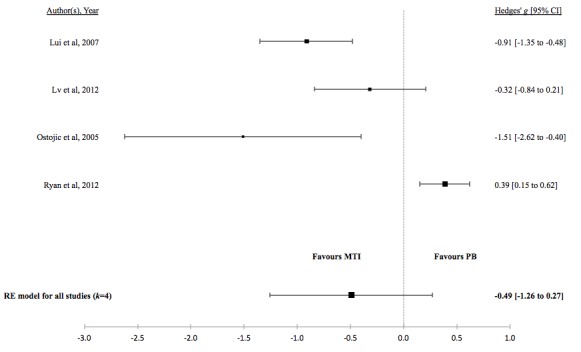
Forest plot of the standardized mean difference in unscheduled visits between MTI and paper-based group. MTI: mobile technology intervention.

**Figure 11:**
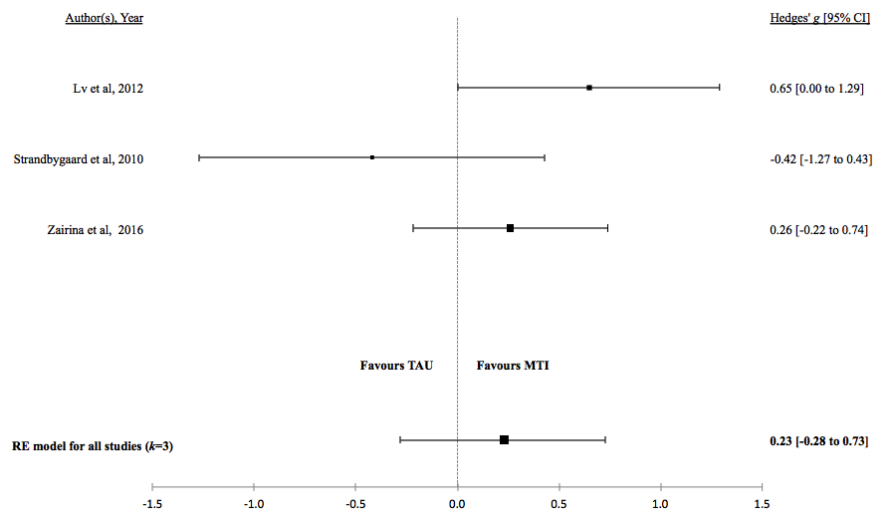
Forest plot of the standardized mean difference in lung function between MTI and standard treatment. MTI: mobile technology intervention.

**Figure 12:**
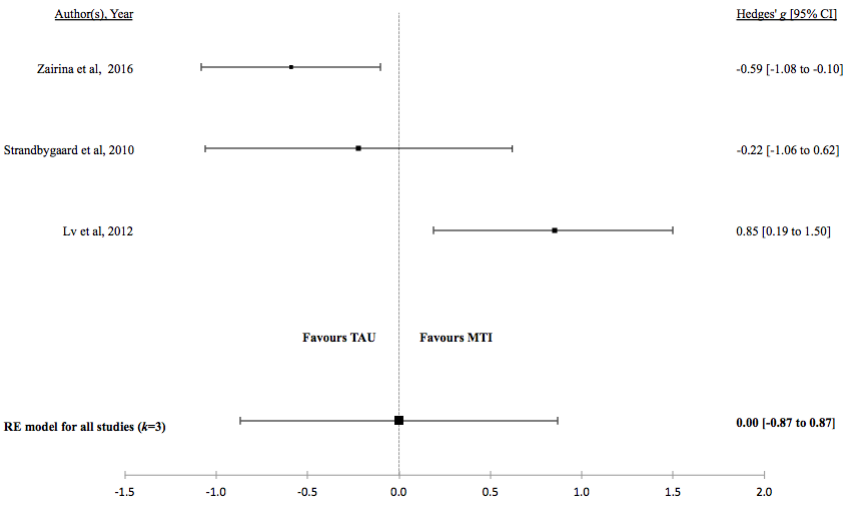
Forest plot of the standardized mean difference in asthma control between MTI and standard treatment. MTI: mobile technology intervention.

**Figure 13:**
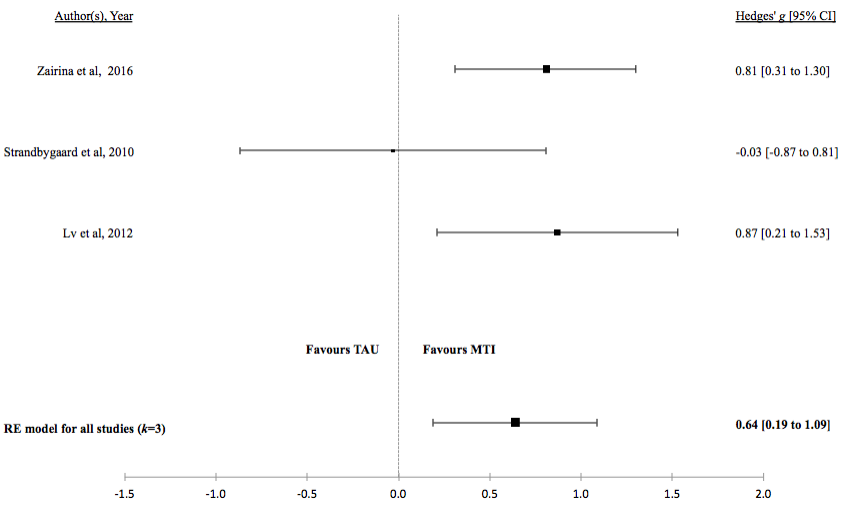
Forest plot of the standardized mean difference in quality of life between MTI and standard treatment. MTI: mobile technology intervention.

**Figure 14:**
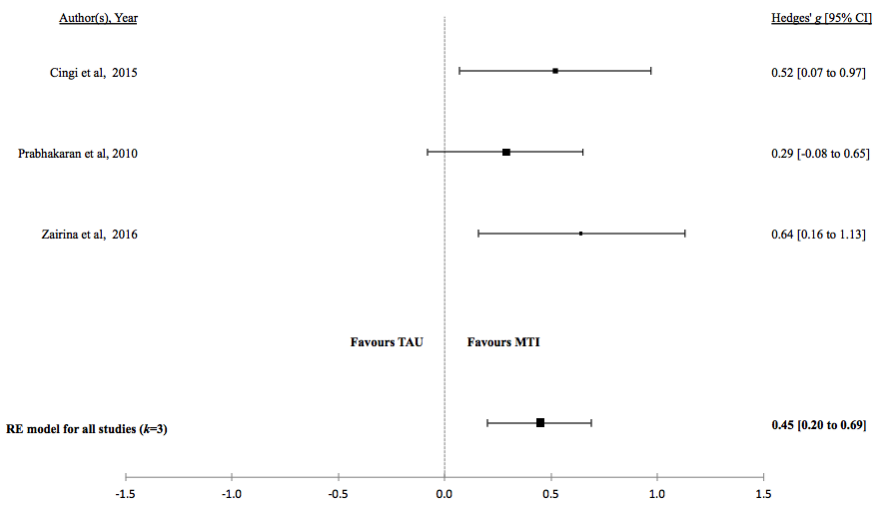
Forest plot of the standardized mean difference in well controlled asthma between MTI and standard treatment. MTI: mobile technology intervention.

**Figure 15:**
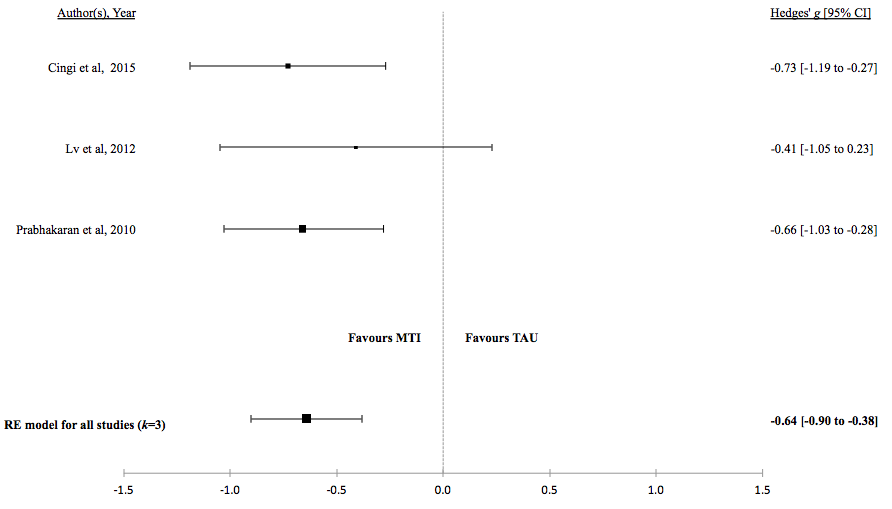
Forest plot of the standardized mean difference in unscheduled visits between MTI and standard treatment. MTI: mobile technology intervention.

There was no evidence for a difference in mean lung function, QoL, asthma control, or percentage of unscheduled visits in studies comparing MTI with paper-based monitoring (Figures 7-10). Similarly, there was no evidence for a difference in mean lung function or asthma control in studies comparing MTI with standard treatment (Figures 11 and 12, respectively). However, there was evidence for a significantly higher standardized mean QoL as well as lower mean percentage of unscheduled visits and more well-controlled asthma in studies comparing MTI with standard treatment (Figures 13-15)

**Table 4:** Hedges *g* and tests of heterogeneity of mobile technology intervention (MTI) for adherence.

Adherence		*k* ^a^	N^b^	Hedges *g* (95%CI^c^)	*P* ^d^	*Q* ^e^	*P* ^f^	*I*^2g^(95% CI)
**MTI vs Paper-based**									
	Medication	4	450	0.16 (-0.03 to 0.34)	.10	1.08	.78	<.01 (<.01-72.42)
	Symptoms	2	136	-0.11 (-0.45 to 0.22)	.51	0.22	.64	<.01 (<.01-99.55)
**MTI vs Standard treatment**									
	Medication	3	169	0.63 (0.31 - 0.94)	<.001	0.53	.77	<.01 (<.01-89.65)

^a^Number of studies.

^b^Total sample size across included studies.

^c^95% CIs around the Hedges *g* effect size.

^d^*P* value of Hedges *g* effect size.

^e^Test of heterogeneity.

^f^*P* value of test for heterogeneity.

^g^Percentage of total variability due to heterogeneity.

**Table 5:** Hedges *g* and tests of heterogeneity of mobile technology intervention (MTI) for clinical outcomes.

Outcome		*k* ^a^	N^b^	Hedges *g* (95%CI^c^)	*P* ^d^	*Q* ^e^	*P* ^f^	*I*^2g^(95% CI)
**MTI vs Paper-based**									
	Lung function	3	162	0.16 (-0.28 to 0.60)	.48	3.1	.21	41.63 (<.01- 97.54)
	QoL^h^	3	347	0.33 (-0.08 to 0.74)	.12	6.79	.03	67.93 (<.01-99.04)
	Asthma control	2	335	0.16 (−0.26 to 0.57)	.46	2.24	.14	55.28 (<.01-99.56)
	Unscheduled visits	4	443	-0.49 (-1.26 to 0.27)	.21	35.5	<.001	90.51 (67.49-99.4)
**MTI vs Standard Treatment**									
	Lung function	3	133	0.23 (-0.28 to 0.73)	.38	3.84	.15	46.15 (<.01-99.07)
	QoL^h^	3	133	0.64 (0.19 - 1.08)	.01	3.29	.19	31.33 (<.01-98.88)
	Asthma control	3	133	0.00 (-0.87 to 0.87)	>.99	11.79	.002	81.27 (33.23-99.50)
	Well controlled	3	273	0.45 (0.20 - 0.69)	<.001	1.43	.49	<.01 (<.01-96.19)
	Unscheduled visits	3	248	-0.64 (-0.90, to 0.38)	<.001	0.66	.72	<.01 (<.01-94.57)

^a^Number of studies.

^b^Total sample size across included studies.

^c^95% CIs around the hedges *g* effect size.

^d^*P* value of Hedges *g* effect size.

^e^Test of heterogeneity.

^f^*P* value of test for heterogeneity.

^g^Percentage of total variability due to heterogeneity.

^h^QoL: Quality of Life.

#### MTI and Publication Bias: Funnel Plots

Across the relationships, symmetry of funnel plots was difficult to assess due to the small number of studies (ranging from *N*=2 -6, mean = 3). The number of studies falling outside of the funnel varied from 0 to 2 (refer to Multimedia Appendix 5 for funnel plots). Two of the 4 relationships with studies outside of the funnel (unscheduled visits between MTI and paper-based as well as asthma control between MTI and standard treatment) also had high heterogeneity, indicating that moderators may be present.

### Moderation of BCT on Adherence and Clinical Outcomes

Tables 6-8 show the results of the moderator analyses. Overall, there was not adequate evidence to determine the type or number of BCT as moderators of adherence or clinical outcomes in studies comparing MTI with standard treatment. However, although the effect of number of BCT used did not reach statistical significance, it did fully explain the variation in QoL.

**Table 6:** Categorical moderation analysis for within group behavior change technique (BCT) type on relationship between mobile technology intervention, adherence, and clinical outcomes.

Outcome	BCT Type	*k* ^a^	Hedges *g* (95%CI)	*P* ^b^
Adherence	Other	1	0.54 (0.15-0.94)	.007
	Prompt Only	2	0.78 (0.25-1.30)	.004
Lung Function	Monitor + feedback	1	0.26 (−1.10-1.62)	.71
	Prompt Only	2	0.17 (−0.62 to 0.97)	.78
QoL^c^	Monitor + feedback	1	0.81 (−0.31 to 1.91)	.15
	Prompt Only	2	0.46 (−0.42 to 1.34)	.30
Asthma Control	Monitor + feedback	1	-0.59 (−1.95 to 0.77)	.40
	Prompt Only	2	0.35 (−0.7 to 1.39)	.51
Well Controlled	Monitor + feedback	2	0.43 (0.09-0.77)	.01
	Monitor Only	1	0.52 (−0.52 to 0.70)	.05

^a^Number of studies.

^b^*P* value of Hedges *g* effect size.

^c^QoL: Quality of Life.

**Table 7:** Categorical moderation analysis for between group behavior change technique (BCT) type on relationship between mobile technology intervention, adherence, and clinical outcomes.

Outcome	*k* ^a^	BCT Type		Other moderators		*I*^2^(95% CI)^f^
		*QM* ^b^	*P* ^c^	*QE* ^d^	*P* ^e^	
Adherence	3	0.48	.49	0.05	.83	<.01 (<.01-97.92)
Lung function	3	0.02	.90	3.84	.05	73.98 (<.01-99.85)
QoL^g^	3	0.23	.63	2.72	.10	63.29 (<.01-99.85)
Asthma control	3	1.14	.29	3.82	.05	73.80, (<.01-99.85)
Well controlled	3	0.08	.78	1.30	.25	23.07 (<.01-99.92)
Unscheduled visits^h^	3					

^a^Number of studies.

^b^Omnibus test of moderator (BCT Type).

^c^*P* value of test of moderator (BCT Type).

^d^Omnibus Test of other moderators not considered in model.

^e^*P* value of test for other moderators.

^f^Percentage of total variability due to heterogeneity.

^g^QoL: Quality of Life.

^h^Mixed effects model would not fit as there was only one study per BCT Type, in the analysis.

**Table 8:** Continuous moderation analysis for number of behavior change technique (BCT) on relationship between mobile technology intervention, adherence, and clinical outcomes.

Outcome	*k* ^a^	Hedges *g* (95%CI)	Test of BCTT^b^#		Test for other moderators		*I*^2^(95% CI)^g^
			*P* ^c^	*R* ^d^	*QE* ^e^	*P* ^f^	
Adherence	3	0.07 (−0.30 to 0.44)	.72	NA	0.40	.53	<.01 (<.01-99.75)
Lung Function	3	0.24 (−0.23 to 0.72)	.32	.00	2.39	.12	58.14 (<.01->99.90)
QoL^h^	3	0.26 (−0.026 to 0.59)	.11	100	0.73	.39	<0.01 (<.01-99.87)
Asthma Control	3	−0.04 (−1.01 to 0.92)	.93	.00	10.65	.001	90.61 (52.83-99.80)
Well Controlled	3	−0.09 (−0.70 to 0.52)	.78	NA	1.30	.25	23.07 (<.01-99.92)
Unscheduled Visits	3	−0.03 (−0.56 to 0.49)	.90	NA	0.64	.42	<.01 (<.01-99.85)

^a^Number of studies.

^b^BCTT: Behavior change technique taxonomy

^c^*P* value of Hedges *g* effect size.

^d^Amount of variation explained by the moderator.

^e^Omnibus Test of other moderators not considered in model.

^f^*P* value of test for other moderators.

^g^Percentage of total variability due to heterogeneity.

^h^QoL: Quality of Life.

### Moderation of Engagement on Adherence and Clinical Outcomes

Only 1 study provided data on engagement for studies comparing MTI with standard treatment [33], and 2 studies comparing MTI with paper-based monitoring [29,31]. Therefore, there were insufficient data to determine if engagement was a moderator between MTI and adherence or clinical outcomes. However, there were sufficient data to test the effect of MTI on attrition using a random effects model, although caution is needed as this was a secondary analysis not included in the initial plan. For what it is worth, there was no evidence for a difference in the standardized mean attrition in studies when comparing MTI with paper-based monitoring and standard treatment (Multimedia Appendix 6).

### Narrative Review

A narrative review was undertaken on the 2 studies that had insufficient data for the meta‐analysis (refer to Multimedia Appendix 3 for the study characteristics). Both studies examined the effect of MTI on improving lung function and quality of life compared with standard treatment [36,37]. Consistent with findings from the meta-analysis, 1 of the 2 studies reporting on QoL found higher levels using MTI compared with standard treatment [36]. Unlike the findings of the meta-analysis, both studies reported on lung function and found improved levels using MTI compared with standard treatment [36,37].

## Discussion

### Principal Findings

The aim of this systematic review and meta-analysis was to determine the efficacy of MTI for asthma self-management. The findings support the effects of MTI for improving adherence and clinical outcomes in asthma [15,21] if compared with standard treatment. Furthermore, these results are consistent with the relationship between MTI, adherence, and clinical outcomes in chronic illness [15] and provide an evidence base for the content of MTI. However, more research is required to determine the role of BCT and engagement in MTI.

The current review found that MTI had a moderate effect on improving medication adherence when compared with standard treatment. Further, the current review suggests that MTI indeed do have a large effect on improving QoL and a moderate effect on individuals achieving well-controlled asthma and fewer unscheduled visits. This is in contrast to previous findings by Tran and colleagues [21] that MTI does not improve clinical outcomes. Consistent with their findings, this review found no evidence of a difference in mean lung function. In addition, the findings in this review are consistent with previous research on chronic illnesses in general that found positive changes in clinical outcomes when using MTI [15], as well as a positive association between medication adherence and improved clinical outcomes for individuals with asthma [5].

Although study duration was identified as a potential limitation to finding change in clinical outcomes [21], the current review noted similar duration in all studies, of around 3 months. The current findings are an advance on previous attempts at systematic reviews of MTI, as it features increased power due to more included studies and by excluding interventions based on telephone-call monitoring, thereby showing clearer effects of MTI. The findings also support claims by physicians working with individuals with asthma who proposed that MTI would improve the quality of care [14]. However, longer duration studies would certainly be valuable. Clinically relevant improvements in QoL, asthma control, and lung function between 3 and 12 months have been found when using Internet-based self-management of asthma compared with standard treatment over 12 months [42], which suggests extending the duration of MTI studies. Furthermore, improvements in QoL and asthma control from Internet-based self-management have been sustained for up to 1.5 years after support has ceased [43].

However, a different picture emerges when MTI effects are compared with paper-based monitoring. Here, the current review found no evidence of a difference in medication or symptom or diary adherence or clinical outcomes.

This suggests 2 indirect conclusions: First, given MTI resulted in improved adherence and clinical outcomes over standard treatment, it suggests that providing instruction on behavior and information about health consequences in itself is not sufficient to improve adherence and consequent health benefits in individuals with asthma. This is consistent with previous findings of a similar improvement in asthma knowledge and inhalation technique between Internet-based self-management and standard treatment over 12 months, concluding that baseline meetings can trigger improvements in these areas without the need for further education [42]. Second, given that there was no difference between MTI and paper-based monitoring, it could suggest improvements in medication adherence and clinical outcomes can be made by providing some feature to monitor medication or symptoms in either electronic or conventional paper form.

As in previous reviews [18], only few of the included studies reported engagement, and when reported at all was widely variable. There was insufficient detail to undertake a formal analysis of engagement, and a secondary analysis looking at individual dropout from the study found no evidence of a difference between MTI and paper-based or standard treatment. However, as MTI are probably an acceptable medium, the fact that they are more portable and potentially more accurate than a paper diary [11-13] points to their potential to help people manage and cope with their asthma.

### Limitations and Future Research

Similar to previous reviews supporting the efficacy of MTI in supporting self-management of chronic illnesses in general [15], this review provides strong evidence for at least short-term efficacy of MTI for asthma management. This supports the need for longer duration studies to identify not just efficacy, but also cost effectiveness, client and physician safety, and the specific constituent behaviour change techniques within the mobile technology intervention that really matters most. Consistent with previous research [15], there was much variation in the study designs and intervention characteristics in this review, making it difficult to draw clear associations between the individual effects of the MTI, the specific study design, and included BCT.

Concerning study quality, small sample size and poor recruitment appear to be a consistent issue in research on MTI and may lead to a lack of generalizability of findings to the broader asthma population [15]. The asthma population varies in regard to asthma severity, health literacy, ability for self-management, and preference for mobile management [44], which makes generalizing findings from single studies and even reviews difficult. The exclusion of individuals with comorbidities also needs addressing as these are frequent in older individuals [1], and this may be contributing to the exclusion of older individuals within MTI research. Furthermore, as recent research [45] has shown that up to one-third of people with physician-diagnosed asthma in the past 5 years do not have current asthma; future treatment evaluations need to establish current asthma objectively, since treatment is unlikely to be effective in people without diagnosed current asthma. For this study, this means that we cannot rule out that people without diagnosed current asthma received treatment which in turn showed no effects.

In addition, this review is limited by the degree to which the included studies reported a theoretical basis for their intervention and subsequently based their interventions on this theory. Internet and mobile-based interventions for promoting health-behavior change have been found to be more efficacious when incorporating more extensive use of theory, BCT, and using text messages as mode of delivery [46]. However, current MTI often lack a theoretical basis underlying the MTI content [15]. In this review, less than half of the included studies provided a theoretical basis for their MTI, and no study specified the BCT used. Thus, the classification of BCT according to a current taxonomy [16] is based only on the information provided in the studies, and we cannot confirm the authors’ original intention.

Two studies in this review found evidence to support changes in medication adherence alongside changes in the individual’s perception of asthma [35,41]. Illness perceptions play a role in asthma self-management [9], with perceived changes to beliefs around duration, control, and severity of asthma associated with the use of MTI with self-monitoring [11,13]. If MTI with self-monitoring indirectly changes asthma perceptions then this validates the Common Sense Model of Illness [10] as a theoretical framework for asthma MTI. Further exploration is warranted on whether targeting illness perceptions alone is sufficient for lasting behavior change and highlights the important related question of defining whom to target with which behaviour change technique. One of the included studies found targeting faulty illness perceptions through MTI increased medication adherence [35]. Paradoxically, this study also found those with higher adherence were less likely to remain in the study [35], suggesting that only people with partially or uncontrolled asthma due to faulty illness perceptions would benefit from this type of MTI.

Another study found individuals with milder asthma were more likely to withdraw from paper-based monitoring than standard treatment or MTI [30]. This may suggest that MTI with monitoring is more attractive than paper-based in those with milder severity. Given the current guidelines recommending action plans for asthma management, an MTI that can store and activate action plans would be suitable for any level of severity, but presumably those at most need at the more severe end of the range. Action plans are most effective at improving clinical outcomes when two to four action points are included based on asthma symptoms or peak expiratory flows [4]. The addition of inhalation devices to electronic monitoring devices, perhaps via Bluetooth, has been proposed to record inhalation use data as well as inhalation technique [44]. However, increasing the complexity of the mobile app will also lead to increases in cost and risk to the individual with asthma and the physician, if not adequately tested [44,47].

Perhaps the main limitation of this review was the small number of included studies, which meant that the power of the tests was relatively low, with a high chance of Type-2 statistical errors [24]. On the other hand, the fact that differences were found at all indicates stable effects. Tests of heterogeneity and effect sizes both produced large confidence intervals, again reflecting imprecise estimates. Furthermore, tests of publication bias could not be performed. To validate the findings of this review and extend the ability to do robust analyses before public use, longer and larger studies are required that incorporate a theoretical basis and behavior change techniques [46], and that follow guidelines for reporting MTI [48].

### Implications and Conclusion

MTI for asthma management can improve medication adherence and quality of life, decrease unscheduled visits, and increase the likelihood of achieving well-controlled asthma compared with standard treatment alone. In addition, MTI appear to be equally as efficacious as paper-based monitoring at achieving higher medication adherence and clinical outcomes. Better reporting of BCT and further research into long-term efficacy of MTI for adherence behavior and clinical outcomes is needed to create an evidence base for specific behaviour change techniques that best support individuals with asthma.

Finally, implementation of mobile technology interventions poses a challenge: Physicians’ concerns regarding increased time and resource demands, accuracy of information, physician liability, and patient confidentiality [14] must be addressed. Furthermore, as MTI are being used to make critical decisions, they must be tested for accuracy and reliability to reduce harm to the individual and potential liability for the physician [47]. Increased involvement of the physician in the development and testing phases of the MTI as well as assessment of privacy issues, and improvements in regulation and safety checks by regulatory bodies and the development of a risk assessment framework for medical mobile app is warranted [44,47].

## Appendix

Multimedia Appendix 1 Search strategy.

Multimedia Appendix 2 Studies excluded during full-text review.

Multimedia Appendix 3 Extracted characteristics of studies in meta-analysis and narrative review.

Multimedia Appendix 4 Risk of bias analysis.

Multimedia Appendix 5 Funnel plots.

Multimedia Appendix 6 Attrition analysis.

## Abbreviations

BCT: Behavior change technique
BCTT: Behavior change technique taxonomy
MTI: mobile technology intervention
MTI-App: MTI with mobile app platform
MTI-SMS: MTI with short messaging service platform
QoL: Quality of Life

## References

[1] Bateman ED, Hurd SS, Barnes PJ, Bousquet J, Drazen JM, FitzGerald M, Gibson P, Ohta K, O'Byrne P, Pedersen SE, Pizzichini E, Sullivan SD, Wenzel SE, Zar HJ (2008). Global strategy for asthma management and prevention: GINA executive summary. Eur Respir J.

[2] (2015). Australian Bureau of Statistics.

[3] (2015). Australian Asthma Foundation.

[4] Gibson PG, Powell H (2004). Written action plans for asthma: an evidence-based review of the key components. Thorax.

[5] Lindsay JT, Heaney LG (2013). Nonadherence in difficult asthma - facts, myths, and a time to act. Patient Prefer Adherence.

[6] Reddel H, Sawyer S, Flood P, Everett P, Peters M (2014). Patterns of asthma control and inhaled corticosteroid (ICS) use in Australians living with asthma. Respirology.

[7] Correll PK, Poulos LM, Ampon R, Reddel HK, Marks GB (2015). Australian Institute of Health and Welfare.

[8] Kandane-Rathnayake RK, Matheson MC, Simpson JA, Tang ML, Johns DP, Mészáros D, Wood-Baker R, Feather I, Morrison S, Jenkins MA, Giles GG, Hopper J, Abramson MJ, Dharmage SC, Walters EH (2009). Adherence to asthma management guidelines by middle-aged adults with current asthma. Thorax.

[9] Horne R, Weinman J (2002). Self-Regulation and self-management in asthma: exploring the role of illness perceptions and treatment beliefs in explaining non-adherence to preventer medication. Health Psychol.

[10] Leventhal H, Meyer D, Nerenz D, Rachman S (1980). The common sense representation of illness danger. Medical Psychology, Volume II.

[11] Cleland J, Caldow J, Ryan D (2007). A qualitative study of the attitudes of patients and staff to the use of mobile phone technology for recording and gathering asthma data. J Telemed Telecare.

[12] Fonseca JA, Costa-Pereira A, Delgado L, Fernandes L, Castel-Branco MG (2006). Asthma patients are willing to use mobile and web technologies to support self-management. Allergy.

[13] Anhøj J, Møldrup C (2004). Feasibility of collecting diary data from asthma patients through mobile phones and SMS (short message service): response rate analysis and focus group evaluation from a pilot study. J Med Internet Res.

[14] Martinasek MP, Panzera AD, Schneider T, Lindenberger JH, Bryant CA, McDermott RJ, Couluris M (2011). Benefits and barriers of pediatric healthcare providers toward using social media in asthma care. Am J Health Behav.

[15] Hall AK, Cole-Lewis H, Bernhardt JM (2015). Mobile text messaging for health: a systematic review of reviews. Annu Rev Public Health.

[16] Michie S, Richardson M, Johnston M, Abraham C, Francis J, Hardeman W, Eccles MP, Cane J, Wood CE (2013). The behavior change technique taxonomy (v1) of 93 hierarchically clustered techniques: building an international consensus for the reporting of behavior change interventions. Ann Behav Med.

[17] Morrissey EC, Corbett TK, Walsh JC, Molloy GJ (2016). Behavior change techniques in apps for medication adherence: a content analysis. Am J Prev Med.

[18] Fjeldsoe BS, Marshall AL, Miller YD (2009). Behavior change interventions delivered by mobile telephone short-message service. Am J Prev Med.

[19] Hamine S, Gerth-Guyette E, Faulx D, Green BB, Ginsburg AS (2015). Impact of mHealth chronic disease management on treatment adherence and patient outcomes: a systematic review. J Med Internet Res.

[20] Marcano Belisario JS, Huckvale K, Greenfield G, Car J, Gunn LH (2013). Smartphone and tablet self management apps for asthma. Cochrane Database Syst Rev.

[21] Tran N, Coffman JM, Sumino K, Cabana MD (2014). Patient reminder systems and asthma medication adherence: a systematic review. J Asthma.

[22] Moher D, Liberati A, Tetzlaff J, Altman DG (2009). Preferred reporting items for systematic reviews and meta-analyses: the PRISMA statement. PLoS Med.

[23] Malhotra S, Musgrave SD, Pinnock H, Price D, Ryan DP (2012). The challenge of recruiting in primary care for a trial of telemonitoring in asthma: an observational study. Pragmat Obs Res.

[24] Higgins JP, Green S (2011). Cochrane Handbook for Systematic Reviews of Interventions Version 5.1.0.

[25] Durlak JA (2009). How to select, calculate, and interpret effect sizes. J Pediatr Psychol.

[26] R Core Team (2011). R-project.

[27] Hedges LV, Vevea JL (1998). Fixed- and random-effects models in meta-analysis. Psychological Methods.

[28] Cingi C, Yorgancioglu A, Cingi CC, Oguzulgen K, Muluk NB, Ulusoy S, Orhon N, Yumru C, Gokdag D, Karakaya G, Çelebi Ş, Çobanoglu HB, Unlu H, Aksoy MA (2015). The “physician on call patient engagement trial” (POPET): measuring the impact of a mobile patient engagement application on health outcomes and quality of life in allergic rhinitis and asthma patients. Int Forum Allergy Rhinol.

[29] Liu W, Huang C, Wang C, Lee K, Lin S, Kuo H (2011). A mobile telephone-based interactive self-care system improves asthma control. Eur Respir J.

[30] Lv Y, Zhao H, Liang Z, Dong H, Liu L, Zhang D, Cai S (2012). A mobile phone short message service improves perceived control of asthma: a randomized controlled trial. Telemed J E Health.

[31] Ostojic V, Cvoriscec B, Ostojic SB, Reznikoff D, Stipic-Markovic A, Tudjman Z (2005). Improving asthma control through telemedicine: a study of short-message service. Telemed J E Health.

[32] Ryan D, Price D, Musgrave SD, Malhotra S, Lee AJ, Ayansina D, Sheikh A, Tarassenko L, Pagliari C, Pinnock H (2012). Clinical and cost effectiveness of mobile phone supported self monitoring of asthma: multicentre randomised controlled trial. BMJ.

[33] Prabhakaran L, Chee WY, Chua KC, Abisheganaden J, Wong WM (2010). The use of text messaging to improve asthma control: a pilot study using the mobile phone short messaging service (SMS). J Telemed Telecare.

[34] Zairina E, Abramson MJ, McDonald CF, Li J, Dharmasiri T, Stewart K, Walker SP, Paul E, George J (2016). Telehealth to improve asthma control in pregnancy: a randomized controlled trial. Respirology.

[35] Petrie KJ, Perry K, Broadbent E, Weinman J (2012). A text message programme designed to modify patients' illness and treatment beliefs improves self-reported adherence to asthma preventer medication. Br J Health Psychol.

[36] Yun T, Arriaga R (2013). A text message a day keeps the pulmonologist away. http://dl.acm.org/citation.cfm?id=2466233.

[37] Yun T, Joeng H, Hill T, Lesnick B, Brown R, Abowd G (2012). Using SMS to provide continuous assessment and improve health outcomes for children with asthma. http://dl.acm.org/citation.cfm?id=2110432.

[38] Rosenstock IM (1974). Historical origins of the health belief model. Health Educ Behav.

[39] Petrie KJ, Weinman J (2006). Why illness perceptions matter. Clin Med (Lond).

[40] Glasziou P, Irwig L, Mant D (2005). Monitoring in chronic disease: a rational approach. BMJ.

[41] Strandbygaard U, Thomsen SF, Backer V (2010). A daily SMS reminder increases adherence to asthma treatment: a three-month follow-up study. Respir Med.

[42] van der Meer V, Bakker MJ, van den Hout WB, Rabe KF, Sterk PJ, Kievit J, Assendelft WJ, Sont JK, SMASHING (Self-Management in Asthma Supported by Hospitals‚ ICT‚ NursesGeneral Practitioners) Study Group (2009). Internet-based self-management plus education compared with usual care in asthma: a randomized trial. Ann Intern Med.

[43] van Gaalen JL, Beerthuizen T, van der Meer V, van Reisen P, Redelijkheid GW, Snoeck-Stroband JB, Sont JK (2013). Long-term outcomes of internet-based self-management support in adults with asthma: randomized controlled trial. J Med Internet Res.

[44] van Boven JF, Trappenburg JC, van der Molen T, Chavannes NH (2015). Towards tailored and targeted adherence assessment to optimise asthma management. NPJ Prim Care Respir Med.

[45] Aaron SD, Vandemheen KL, FitzGerald JM, Ainslie M, Gupta S, Lemière C, Field SK, McIvor RA, Hernandez P, Mayers I, Mulpuru S, Alvarez GG, Pakhale S, Mallick R, Boulet L, Canadian Respiratory Research Network (2017). Reevaluation of diagnosis in adults with physician-diagnosed asthma. JAMA.

[46] Webb TL, Joseph J, Yardley L, Michie S (2010). Using the internet to promote health behavior change: a systematic review and meta-analysis of the impact of theoretical basis, use of behavior change techniques, and mode of delivery on efficacy. J Med Internet Res.

[47] Lewis TL, Wyatt JC (2014). mHealth and mobile medical apps: a framework to assess risk and promote safer use. J Med Internet Res.

[48] Agarwal S, LeFevre AE, Lee J, L'Engle K, Mehl G, Sinha C, Labrique A, WHO mHealth Technical Evidence Review Group (2016). Guidelines for reporting of health interventions using mobile phones: mobile health (mHealth) evidence reporting and assessment (mERA) checklist. BMJ.

