# Supplementary material for: Mobile Technology Interventions for Asthma Self-Management: Systematic Review and Meta-Analysis
Source: JMIR Mhealth Uhealth. 2017 May 2;5(5):e57. doi: 10.2196/mhealth.7168 (PMC5434254; doi:10.2196/mhealth.7168)
Supplement: Multimedia Appendix 1 [file mhealth_v5i5e57_app1.pdf]

**Proquest PsychInfo**
TI,AB,SU(ASTHMA*) AND TI,AB,SU(MOBILE* OR TELE* OR PHONE OR SMS OR MULTIMEDIA OR MULTI*MEDIA OR TEXT* OR WIRELESS OR MESSAGE* OR MMS OR M*HEALTH OR E*HEALTH OR ANDROID OR I-P* OR SM) AND TI,AB,SU(INTERVENTION OR RCT OR TRIAL OR CONTROL* OR GROUP* OR RANDOM*) AND TI,AB,SU(ADHERE* OR MONITOR* OR RECORD* OR ENTER*)
Limits: peer review, English, year: until June 30, 2016
**Medline (PubMed)**
((ASTHMA*[Title/Abstract]) AND (MOBILE[Title/Abstract] OR TELE*[Title/Abstract] OR *PHONE[Title/Abstract] OR SMS[Title/Abstract] OR MULTIMEDIA[Title/Abstract] OR MULTI*MEDIA[Title/Abstract] OR TEXT*[Title/Abstract] OR WIRELESS[Title/Abstract] OR MESSAGE*[Title/Abstract] OR MMS[Title/Abstract] OR M*HEALTH[Title/Abstract] OR E*HEALTH[Title/Abstract] OR ANDROID[Title/Abstract] OR I-P*[Title/Abstract] OR SMART*[Title/Abstract]) AND (INTERVENTION[Title/Abstract] OR CONTROL[Title/Abstract] OR RCT[Title/Abstract] OR TRIAL[Title/Abstract] OR GROUP*[Title/Abstract] OR RANDOM*[Title/Abstract]) AND (ADHERE*[Title/Abstract] OR MONITOR*[Title/Abstract] OR RECORD*[Title/Abstract] OR ENTER*[Title/Abstract]))
Limits: English, year: until June 30, 2016
**Scopus**
TITLE-ABS-KEY (ASTHMA*) AND TITLE-ABS-KEY (MOBILE* OR TELE* PHONE OR SMS OR MULTIMEDIA OR MULTI*MEDIA OR TEXT* OR WIRELESS OR MESSAGE* OR MMS OR M*HEALTH OR E*HEALTH OR ANDROID OR I-P*OR SMART*) AND TITLE-ABS-KEY (INTERVENTION OR RCT OR TRIAL OR RANDOM* OR CONTROL* OR GROUP*) AND TITLE-ABS-KEY (ADHERE* OR MONITOR* OR RECORD* OR ENTER*)
Limits: English, year: until June 30, 2016
**Web of Science**
(ASTHMA* AND (MOBILE* OR TELE* OR PHONE OR SMS OR MULTIMEDIA OR MULTI*MEDIA OR TEXT* OR WIRELESS OR MESSAGE* OR MMS OR M*HEALTH OR E*HEALTH OR ANDROID OR I-P*OR SMART*) AND (ADHERE* OR MONITOR* OR RECORD* OR ENTER*) AND (INTERVENTION OR RCT OR TRIAL OR RANDOM* OR CONTROL* OR GROUP*))
Limits: article or review, English, year: until June 30, 2016
**ProQuest Dissertations and Theses Global**
TI,AB,SU(ASTHMA*) AND TI,AB,SU(MOBILE* OR TELE* OR PHONE OR SMS OR

MULTIMEDIA OR MULTI*MEDIA OR TEXT* OR WIRELESS OR MESSAGE* OR MMS OR M*HEALTH OR E*HEALTH OR ANDROID OR I-P* OR SM) AND TI,AB,SU(INTERVENTION OR RCT OR TRIAL OR CONTROL* OR GROUP* OR RANDOM*) AND TI,AB,SU(ADHERE* OR MONITOR* OR RECORD* OR ENTER*)
Limits: English, year: until June 30, 2016
**Clinicaltrials.gov**
ASTHMA* AND (MOBILE* OR TELE* OR PHONE OR SMS OR MULTIMEDIA OR MULTI*MEDIA OR TEXT* OR WIRELESS OR MESSAGE* OR MMS OR M*HEALTH OR E*HEALTH OR ANDROID OR I-P*OR SMART*) AND (ADHERE* OR MONITOR* OR RECORD* OR ENTER*) AND (INTERVENTION OR RCT OR TRIAL)
