# Supplementary material for: Mobile Technology Interventions for Asthma Self-Management: Systematic Review and Meta-Analysis
Source: JMIR Mhealth Uhealth. 2017 May 2;5(5):e57. doi: 10.2196/mhealth.7168 (PMC5434254; doi:10.2196/mhealth.7168)
Supplement: Multimedia Appendix 2 [file mhealth_v5i5e57_app2.pdf]

Excluded articles (n=50)

Summary of reasons:

1. Study was not a Randomized Control Trial (n=13)
2. Intervention was not an Mobile Technology Intervention (n=25)
3. Targeted population was the child and the parent (n=3)
4. There was no control group for the MTI (n= 5)
5. No target behavior outcomes (n=1)
6. Study month or less (n=3)

List of excluded articles:

(1) Study was not a Randomized Control Trial (n=13)

1. Anantharam P, Banerjee T, Sheth A, Thirunarayan K, Marupudi S, Sridharan V, et al., editors. Knowledge-Driven Personalized Contextual mHealth Service for Asthma Management in Children. Proceedings - 2015 IEEE 3rd International Conference on Mobile Services, MS 2015; 2015.
2. Elias P, Rajan NO, McArthur K, Dacso CC. InSpire to promote lung assessment in youth: Evolving the self-management paradigms of young people with asthma. Journal of Medical Internet Research. 2013;15(5). doi: 10.2196/med20.2014.
3. Foster JM, Smith L, Usherwood T, Sawyer SM, Rand CS, Reddel HK. The reliability and patient acceptability of the SmartTrack device: a new electronic monitor and reminder device for metered dose inhalers. The Journal of asthma : official journal of the Association for the Care of Asthma. 2012;49(6):657-62. Epub 2012/06/30. doi: 10.3109/02770903.2012.684253. PubMed PMID: 22741746.
4. Kassem A, Hamad M, El-Moucary C, Neghawi E, Bou Jaoude G, Merhej C, editors. Asthma Care Apps. 2013 2nd International Conference on Advances in Biomedical Engineering, ICABME 2013; 2013.
5. Kwan AM, Fung AG, Jansen PA, Schivo M, Kenyon NJ, Delplanque JP, et al. Personal Lung Function Monitoring Devices for Asthma Patients. Ieee Sensors Journal. 2015;15(4):2238-47. doi: 10.1109/jsen.2014.2373134. PubMed PMID: WOS:000349622800010.
6. Lee HR, Yoo SK, Jung SM, Kwon NY, Hong CS. A Web-based mobile asthma management system. J Telemed Telecare. 2005;11 Suppl 1:56-9. Epub 2005/07/23. doi: 10.1258/1357633054461598. PubMed PMID: 16035995.
7. Licskai CJ, Sands TW, Ferrone M. Development and pilot testing of a mobile health solution for asthma self-management: Asthma action plan smartphone application pilot study. Canadian Respiratory Journal. 2013;20(4):301-6.
8. Moldrup C, Stein J, Sondergaard B. "Patients don't lie"; a view on adherence in asthma. Pharmacy world & science : PWS. 2010;32(6):795-8.
9. Mosnaim G, Li H, Martin M, Richardson D, Belice PJ, Avery E, et al. A tailored mobile health intervention to improve adherence and asthma control in minority adolescents. The journal of allergy and clinical immunology In practice. 2015;3(2):288-90.e1.
10. Rhee H, Allen J, Mammen J, Swift M. Mobile phone-based asthma self-management aid for adolescents (mASMAA): a feasibility study. Patient preference and adherence. 2014;8:63-72.
11. Rhee H, Belyea MJ, Sterling M, Bocko MF. Evaluating the Validity of an Automated Device for Asthma Monitoring for Adolescents: Correlational Design. J Med Internet Res. 2015;17(10):e234.

12. Rhee H, Miner S, Sterling M, Halterman JS, Fairbanks E. The development of an automated device for asthma monitoring for adolescents: methodologic approach and user acceptability. JMIR mHealth and uHealth. 2014;2(2):e27.
13. Wise M, Gustafson DH, Sorkness CA, Molfenter T, Staresinic A, Meis T, et al. Internet telehealth for pediatric asthma case management: integrating computerized and case manager features for tailoring a Web-based asthma education program. Health Promot Pract. 2007;8(3):282-91.

(2) Intervention was not solely Mobile Technology Intervention / Intervention involved the internet, paper, school, modem, palmtop, medication tracker, telephone call, or letter (n=25)

1. Biswas R. SmartInhaler: A Platform for Measurement of Inhaler Usage in Asthma Patients. Rice University, Masters Thesis. 2013.
2. Blanchet KD. Remote Monitoring of Asthma. Telemedicine Journal and E-Health. 2009;15(3):227-30.
3. Clark NM, Shah S, Dodge JA, Thomas LJ, Andridge RR, Little RJA. An Evaluation of Asthma Interventions for Preteen Students. Journal of School Health. 2010;80(2):80-7.
4. Deschildre A, Beghin L, Salleron J, Iliescu C, Thumerelle C, Santos C, et al. Home telemonitoring (forced expiratory volume in 1 s) in children with severe asthma does not reduce exacerbations. European Respiratory Journal. 2012;39(2):290-6.
5. Finkelstein J, Cabrera MR, Hripcsak G. Internet-based home asthma telemonitoring - Can patients handle the technology? Chest. 2000;117(1):148-55.
6. Finkelstein J, Hripcsak G, Cabrera MR. Patients' acceptance of Internet-based home asthma telemonitoring. Proceedings / AMIA Annual Symposium AMIA Symposium. 1998:336-40.
7. Hafner D, Reich K, Matricardi PM, Meyer H, Kettner J, Narkus A. Prospective validation of 'Allergy-Control-SCORE(TM)': a novel symptom-medication score for clinical trials. Allergy. 2011;66(5):629-36.
8. Joseph CL, Havstad SL, Johnson D, Saltzgaber J, Peterson EL, Resnicow K, et al. Factors associated with nonresponse to a computer-tailored asthma management program for urban adolescents with asthma. The Journal of asthma : official journal of the Association for the Care of Asthma. 2010;47(6):667-73.
9. Koufopoulos JT, Conner MT, Gardner PH, Kellar I. A Web-Based and Mobile Health Social Support Intervention to Promote Adherence to Inhaled Asthma Medications: Randomized Controlled Trial. J Med Internet Res. 2016;18(6):e122.
10. Lau AYS, Arguel A, Dennis S, Liaw ST, Coiera E. "Why Didn't it Work?" Lessons From a Randomized Controlled Trial of a Web-based Personally Controlled Health Management System for Adults with Asthma. Journal of Medical Internet Research. 2015;17(12).
11. Malmstrom K, Peszek I, Al B, Lu S, Enright PL, Reiss TF. Quality assurance of asthma clinical trials. Controlled clinical trials. 2002;23(2):143-56.
12. Mancuso CA, Peterson MGE, Gaeta TJ, Fernandez JL, Birkhahn RH, Melniker LA, et al. A Randomized Controlled Trial of Self-Management Education for Asthma Patients in the Emergency Department. Annals of emergency medicine. 2011;57(6):603-12.
13. Merchant RK, Inamdar R, Quade RC. Effectiveness of Population Health Management Using the Propeller Health Asthma Platform: A Randomized Clinical Trial. The journal of allergy and clinical immunology In practice. 2016;4(3):455-63.

14. Patel M, Pilcher J, Pritchard A, Perrin K, Travers J, Shaw D, et al. Efficacy and safety of maintenance and reliever combination budesonide-formoterol inhaler in patients with asthma at risk of severe exacerbations: a randomised controlled trial. The Lancet Respiratory medicine. 2013;1(1):32-42.
15. Patel M, Pilcher J, Reddel HK, Pritchard A, Corin A, Helm C, et al. Metrics of salbutamol use as predictors of future adverse outcomes in asthma. Clinical and experimental allergy : journal of the British Society for Allergy and Clinical Immunology. 2013;43(10):1144-51.
16. Patel M, Pilcher J, Reddel HK, Qi V, Mackey B, Tranquilino T, et al. Predictors of severe exacerbations, poor asthma control, and beta-agonist overuse for patients with asthma. The journal of allergy and clinical immunology In practice. 2014;2(6):751-8.
17. Patel M, Pilcher J, Travers J, Perrin K, Shaw D, Black P, et al. Use of metered-dose inhaler electronic monitoring in a real-world asthma randomized controlled trial. The journal of allergy and clinical immunology In practice. 2013;1(1):83-91.
18. Roman-Rodriguez M, Pardo MG, Lopez LG, Ruiz AU, van Boven J. Enhancing the use of Asthma and COPD Assessment Tools in Balearic Primary Care (ACATIB): a region-wide cluster-controlled implementation trial. NPJ primary care respiratory medicine. 2016;26:16003.
19. Uysal MA, Mungan D, Yorgancioglu A, Yildiz F, Akgun M, Gemicioglu B, et al. Asthma control test via text messaging: could it be a tool for evaluating asthma control? The Journal of asthma : official journal of the Association for the Care of Asthma. 2013;50(10):1083-9.
20. Vollmer WM, Xu MC, Feldstein A, Smith D, Waterbury A, Rand C. Comparison of pharmacy-based measures of medication adherence. Bmc Health Services Research. 2012;12.
21. Voorend-van Bergen S, Vaessen-Verberne AA, Brackel HJ, Landstra AM, van den Berg NJ, Hop WC, et al. Monitoring strategies in children with asthma: a randomised controlled trial. Thorax. 2015;70(6):543-50.
22. Weinberger M, Murray MD, Marrero DG, Brewer N, Lykens M, Harris LE, et al. Effectiveness of pharmacist care for patients with reactive airways disease - A randomized controlled trial. Jama-Journal of the American Medical Association. 2002;288(13):1594-602.
23. Willems DCM, Joore MA, Hendriks JJE, Nieman FHM, Severens JL, Wouters EFM. The effectiveness of nurse-led telemonitoring of asthma: Results of a randomized controlled trial. Journal of Evaluation in Clinical Practice. 2008;14(4):600-9.
24. Willems DCM, Joore MA, Hendriks JJE, van Duurling RAH, Wouters EFM, Severens JL. Process evaluation of a nurse-led telemonitoring programme for patients with asthma. Journal of Telemedicine and Telecare. 2007;13(6):310-7.
25. Zeiger RS, Schatz M, Li QW, Solari PG, Zazzali JL, Chen WS. Real-Time Asthma Outreach Reduces Excessive Short-acting beta(2)-Agonist Use: A Randomized Study. Journal of Allergy and Clinical Immunology-in Practice. 2014;2(4):445-U159.

(3) Targeted population was the child and the parent (n=3)

1. Arga M, Sahbaz H, Bakirtas A, Turktas I, Demirsoy MS. Does self-monitoring by means of symptom diaries improve asthma control in children? The Journal of asthma : official journal of the Association for the Care of Asthma. 2014;51(3):299-305.
2. Jan RL, Wang JY, Huang MC, Tseng SM, Su HJ, Liu LF. An Internet-based interactive telemonitoring system for improving childhood asthma outcomes in Taiwan. Telemedicine Journal and E-Health. 2007;13(3):257-68.

3. Vasbinder EC, Goossens LM, Rutten-van Molken MP, de Winter BC, van Dijk L, Vulto AG, et al. e-Monitoring of Asthma Therapy to Improve Compliance in children (e-MATIC): a randomised controlled trial. The European respiratory journal. 2016.

(4) There was no control group for the MTI (n= 5)

1. de Jongste JC, Carraro S, Hop WC, Baraldi E, Grp CS. Daily Telemonitoring of Exhaled Nitric Oxide and Symptoms in the Treatment of Childhood Asthma. American journal of respiratory and critical care medicine. 2009;179(2):93-7.
2. Mosnaim G, Li H, Martin M, Richardson D, Belice PJ, Avery E, et al. The impact of peer support and mp3 messaging on adherence to inhaled corticosteroids in minority adolescents with asthma: a randomized, controlled trial. The journal of allergy and clinical immunology In practice. 2013;1(5):485-93.
3. Naar-King S, Ellis D, King PS, Lam P, Cunningham P, Secord E, et al. Multisystemic Therapy for high-risk African American adolescents with asthma: A randomized clinical trial. Journal of Consulting and Clinical Psychology. 2014;82(3):536-45.
4. van der Meer V, van Stel HF, Detmar SB, Otten W, Sterk PJ, Sont JK. Internet-based self-management offers an opportunity to achieve better asthma control in adolescents. Chest. 2007;132(1):112-9.
5. van Gaalen JL, Beerthuizen T, van der Meer V, van Reisen P, Redelijkheid GW, Snoeck-Stroband JB, et al. Long-Term Outcomes of Internet-Based Self-Management Support in Adults With Asthma: Randomized Controlled Trial. Journal of Medical Internet Research. 2013;15(9):40-52.

(5) No target behavior or primary outcomes (n=1)

1. Mosnaim GS, Cohen MS, Rhoads CH, Rittner SS, Powell LH. Use of MP3 players to increase asthma knowledge in inner-city African-American adolescents. Int J Behav Med. 2008;15(4):341-6.

(6) Study month or less (n=3)

1. Seid M, D'Amico EJ, Varni JW, Munafo JK, Britto MT, Kercsmar CM, et al. The in vivo adherence intervention for at risk adolescents with asthma: report of a randomized pilot trial. J Pediatr Psychol. 2012;37(4):390-403. Epub 2011/12/15. doi: 10.1093/jpepsy/jsr107. PubMed PMID: 22167121; PubMed Central PMCID: PMCPMC3334534.
2. Meltzer EO, Kelley N, Hovell MF. Randomized, cross-over evaluation of mobile phone vs paper diary in subjects with mild to moderate persistent asthma. The Open Respiratory Medicine Journal,. 2008;2:72-9. doi: 10.2174/1874306400802010072
3. Johnson KB, Patterson BL, Ho YX, Chen Q, Nian H, Davison CL, et al. The feasibility of text reminders to improve medication adherence in adolescents with asthma. Journal of the American Medical Informatics Association : JAMIA. 2016;23(3):449-55. Epub 2015/12/15. doi: 10.1093/jamia/ocv158. PubMed PMID: 26661717; PubMed Central PMCID: PMCPMC4901375.
