# Supplementary material for: Mobile Technology Interventions for Asthma Self-Management: Systematic Review and Meta-Analysis
Source: JMIR Mhealth Uhealth. 2017 May 2;5(5):e57. doi: 10.2196/mhealth.7168 (PMC5434254; doi:10.2196/mhealth.7168)
Supplement: Multimedia Appendix 3 [file mhealth_v5i5e57_app3.pdf]

Table A3.1. Extracted characteristics of included studies in Meta-analysis

Study	Research design	Population & Sample Sizes	Intervention	Intervention effects
Ostojic et al., (2005)	Study: Equal 2 arm RCT (Parallel);<br>Platform: SMS<br>Duration: 16 weeks<br>Interactivity: Daily:<br>Recruitment: Pulmonary clinic;	Participants *N*: 16 (MTI: 8, PB: 8);<br>Demographics: Adults<br>Mean age: 25 years;<br>Female: 44%,<br>Asthma: Moderate for 6 months according to GINA<br>Country Croatia;	PB: paper diary to record PEF measures, medication use, and asthma symptoms. Diary reviewed at end of study.<br>MTI: SMS PEF measures daily to Dr, receive weekly reply of therapy adjustments and recommendations for follow-up. If PEF exacerbated received SMS for office visit.	Outcomes:<br>• Adherence - ICS Dosage – Post Mean (SD): MTI 625 (332) vs. PB 530 (200); *P* = .57<br>• Adherence - PEF – Post %: MTI 66.2 vs. PB 61.3, *P = .88*<br>• Lung Function - FEV_1 % Predicted – Post Mean (SD): MTI 81.25 (17.31) vs. PB 78.25 (21.09); *P* = .57<br>• Unscheduled Visits – MTI 25% vs. 88%<br>• Engagement: 99%<br>• Attrition: MTI 0% vs. PB 0%
Liu et al., (2007)	Study: 2 arm RCT (Parallel);<br>Platform: Mobile App<br>Duration: 6 months<br>Interactivity: Daily<br>Recruited: Outpatient clinic;	Participants *N*: 120 (MTI: 60, TAU: 60);<br>Demographics: Adults<br>Mean age: 52 years;<br>Female: 51%,<br>Asthma: Moderate-severe persistent according to American Thoracic Society,<br>Country Taiwan;	PB: Asthma education, self-management plan, and standard treatment. Record daily PEF and asthma symptoms on a diary. Follow-up every 1 month<br>MTI: same as PI but electronic diary for recording weekly plus based on these measures received daily reply of asthma status and therapy adjustments.	Outcomes:<br>• Adherence - ICS Dosage – Post Mean (SE): MTI 709 (32) vs. PB 630 (39); *P* < .05<br>• Lung Function - FEV_1 % Predicted – Post Mean (SE): MTI 65.2 (3.2) vs. PB 56.5 (2.8); *P* < .05<br>• QoL -SF-12 PCS – post Mean (SE): MTI 45.5 (1.4) vs. PB 40.0 (1.5), *P* < .01<br>• Unscheduled Visits – MTI 4% vs. PB 20%, *P* < .05<br>• Attrition: MTI 28% vs. PB 23%
Prabhakaran et al., (2010)	Study: RCT Study;<br>Platform: SMS<br>Duration: 3 months<br>Interactivity: daily SMS;<br>Recruited: patients admitted to hospital for asthma	Participants *N*: 120 (MTI: 60; TAU: 60);<br>Demographics: 21+ years<br>Mean: 55 years<br>Female: 65%,<br>Asthma: undefined<br>Country Singapore;	TAU: asthma education<br>MTI: SMS for 2 weeks regarding asthma symptoms, If yes, thank. Weekly SMS for 10 weeks regarding ventilation use and report on achieved control. If not achieved contacted for advice and change of appointment if required. If no reply then sent another SMS in evening.	Outcomes:<br>• Well Controlled ACT - %: MTI 62% vs. TAU 49%, *P* = 0.11<br>• Fewer Unscheduled Visits – %: MTI 85% vs. TAU 95%, *P* = .06<br>• Engagement: 82%<br>• Attrition: MTI 3% vs. TAU 5%

Strandbygaard et al. (2010)	Study: RCT Study;<br>Platform: SMS<br>Duration: 3 months<br>Interactivity: daily SMS;<br>Recruited: advertisement in local paper	Participants *N*: 26 (MTI:12; TAU: 14);<br>Demographics: 18-45 years<br>Mean: 32 years<br>Female: 46%,<br>Asthma: undefined mild-severe persistent<br>Country: Denmark	TAU: education on ICS treatment, asthma knowledge, correct inhaler technique<br>MTI: TAU + daily SMS to take meds for 8 weeks at 10am	Outcomes:<br>• Adherence - ICS – Change Mean (95%CI): MTI 3.6 (-8.5, 15.7) vs. TAU -14.2 (-24.2, 4.1); *P* = .02<br>• Lung Function - FEV_1 % Predicted – Change Mean (95%CI): MTI 3.28 (-1.94, 8.50) vs. TAU 7.65 (0.22, 15.07); *P* = .32<br>• QoL - Mini AQLQ – Change Mean (95%CI): MTI 0.57 (0.05, 1.10) vs. TAU 0.59 (0.13, 1.05), *P* = .96<br>• Asthma Control - ACQ – Change Mean (95%CI): MTI 0.57 (0.05, 1.10) vs. TAU 0.59 (0.27, 0.90), *P* = .39<br>• Attrition: MTI 17% vs. TAU 14%
Lv et al., (2012)	Study: RCT Study;<br>Platform: SMS<br>Duration: 3 months<br>Interactivity: Twice Daily;<br>Recruited: Outpatient clinic	Participants *N*: 150 (TAU:50, PB:50, MTI:50);<br>Demographics: 18+ years<br>Mean age: 38 years<br>Female: 42%,<br>Asthma: 3+ months according to GINA<br>Country: China	TAU: asthma education<br>PB: TAU + action plan, monitor PEF and asthma symptom on paper diary), how to adjust plan from data<br>MTI: TAU + daily SMS (10am, 8pm) on asthma management, asthma medications, device use, triggers and how to them, how to make an action plan, and how to handle asthma acute attacks. Send SMS to staff to answer questions.	Outcomes:<br>• Adherence - %: MTI 80% vs. PB 74% vs. TAU 50%; *P* = .11<br>• Lung Function - FEV_1 % Predicted – Change Mean (SD): MTI 11.42 (17.07) vs. PB 14.61 (20.19) vs. TAU 1.62 (8.21); *P* = .07<br>• QoL - AQLQ – Change Mean (SD): MTI 31.4 (30.42) vs. PB 16.52 (21.1) vs. TAU 4.21 (30.98), *P* =.01<br>• Asthma Control - PCAQ-6 - Change Mean (SD): MTI 7.07 (4.44) vs. PB 4.78 (5.77) vs. TAU 3 (5.31), *P* = .046<br>• Unscheduled Visit: MTI 18.3% vs. PB 28.9% vs. 32.7%, *P* = .09<br>• Attrition: MTI 40% vs. PB 46% vs. TAU 72%

Petrie et al., (2012)	Study: RCT Study;<br>Platform: SMS<br>Duration: 18 weeks<br>Interactivity: daily SMS;<br>Recruited: pamphlets with asthma meds and emails of marketing website	Participants *N*: 147 (MTI:73; TAU: 74);<br>Demographics: 16-45 years<br>Mean: ?<br>Female: 68%,<br>Asthma: undefined with adherence issues<br>Country: United Kingdom	Baseline Illness perceptions assessed<br>TAU: Baseline Illness perceptions assessed<br>MTI: SMS for 18 weeks targeted at illness beliefs (~24 texts generated per belief)<br>1-6 weeks: 2 texts per day;<br>7-12 weeks: 1 per day;<br>13-18 weeks: 3 per day.	Outcomes:<br>• Adherence - Post Mean (SD): MTI 57.8 (27.1) vs. TAU 43.2 (26.0); $P = .03$<br>• Attrition: MTI 22% vs. TAU 38%
Ryan et al., (2012)	Study: RCT Study;<br>Platform: Mobile App<br>Duration: 6 months<br>Interactivity: Twice Daily;<br>Recruited: 32 GP databases (2.4% recruitment rate)	Participants *N*: 288 (I: 145, PB: 143);<br>Demographics: 12+ years<br>Mean age: 49 years<br>Female: 63%,<br>Asthma: ACQ ≥ 1.5 (no exacerbation in 3 months)<br>Country: United Kingdom	PB: twice daily paper-based recording of symptoms, drug use, and PEF<br>MTI: exactly the same but via mobile phone with immediate feedback prompting action according to an agreed plan	Outcomes:<br>• Adherence - %increased meds: MTI 59% vs. PB 55%<br>• QoL - mini AQLQ – Change Mean (95%CI): MTI -0.75 (-0.94, -0.57) vs. PB -0.65 (-0.84, -0.46), $P > .05$<br>• Asthma Control - ACQ - Change Mean (95%CI): MTI 0.75 (0.61, 0.89) vs. PB 0.73 (0.57, 0.89), $P > .05$<br>• Well-controlled (%): MTI 21% vs. 22%<br>• Unscheduled Visit: MTI 2% vs. PB 1%<br>• Attrition: MTI 1% vs. PB 1%
Cingi et al., (2015)	Study: RCT Study (parallel);<br>Platform: Mobile App<br>Duration: 12 week<br>Interactivity: Daily;<br>Recruited: Multi-site Pulmonary Research Hospital	Participants *N*: 136 (MTI: 68, TAU: 68);<br>Demographics: 24-41 years<br>Mean age: 33 years<br>Female: 53%,<br>Asthma: Mild to severe persistent asthma according to GINA<br>Country: Turkey	TAU: Educated on use of prescribed medication and ACT. Received mobile application to enter ACT score at start and end of trial. Educated on use of prescribed medication and ACT<br>MTI: Educated on use of prescribed medication and ACT. Received mobile application to record health status, track medication use, send and receive messages, request help and automated reminders.	Outcomes:<br>• Well Controlled asthma = MTI 49% vs. TAU 27%, $P < .05$<br>• Unscheduled Visits = MTI 10.9% vs. TAU 32.1%, $P = .02$<br>• Engagement: median 90 times<br>• Attrition: MTI 12% vs. TAU 57%

Zairina et al., (2016)	Study: RCT Study (parallel);<br>Platform: Mobile App<br>Duration: 24 week<br>Interactivity: Weekly;<br>Recruited: Multi-site (2) Antenatal Hospital Clinic	Participants *N*: 72 (MTI: 36, TAU: 36);<br>Demographics: 18+ years<br>Mean age: 31 years<br>Female: 100%,<br>Asthma: undefined mild to severe asthma<br>Country: Australia	TAU: informed about health consequences of asthma and pregnancy<br>MTI: Monitor symptoms and medication, were provided with action plan, automatic feedback on asthma status	Outcomes:<br>• Lung Function - FEV_1 % Predicted – Change Mean (SE): MTI 4.27 (1.86) vs. TAU 1.54 (1.72); *P* = .29<br>• QoL – mini AQLQ – Change Mean (SE): MTI 0.51(0.16) vs. TAU 0.22 (0.15); *P* = .002<br>• Asthma Control - ACQ – Change Mean, SE: MTI 0.30 (0.11) vs. TAU 0.06 (0.10); *P* = .02<br>• Well Controlled asthma = MTI 82% vs. TAU 58%<br>• Unscheduled Visits = MTI 0% vs. TAU 2.9%<br>• Attrition: MTI 11% vs. TAU 3%

Abbreviations:

ACQ: Asthma Control Questionnaire
ACT: Asthma Control Test
AQLQ: Asthma Quality of Life Questionnaire
FEF: Forced Expiratory Flow
FEV_1: Forced Expiratory Volume in 1 second
GINA: Global Initiative for Asthma
ICS: Inhaled Corticosteroids
Mobile App: Mobile Application
MTI: Mobile Technology Intervention
PAQLQ: Pediatric Asthma Quality of Life Questionnaire
PB: Paper-based monitoring
PCAQ: Perceived Control of Asthma Questionnaire
PedsQL: Pediatric Quality of Life
PEFR: Peak Expiratory Flow Rate Meter
QoL: Quality of Life
RCT: Randomised Control Trial
SD: Standard Deviation:
SE: Standard Error:
SF-12: Short form (SF)-12 Questionnaire
SMS: Short Message Service

TAU: Treatment as Usual
95%CI: 95% Confidence Interval

Table A3.2. Extracted characteristics of included studies in narrative review

Study	Research design	Population & Sample Sizes	Intervention	Primary and Secondary Outcomes
Yun et al. (2012)	Study: RCT Study;<br>Platform: SMS<br>Duration: 3 months<br>Interactivity: SMS;<br>Recruited: Private paediatric asthma clinic	Participants *N*: 30 (10 each);<br>Demographics: 10-16 years<br>Mean: 14 years<br>Female: 50%<br>Asthma: undefined mid-severe persistent<br>Country: United States of America	TAU: No SMS<br>MTI-Symptom: Text re symptom awareness every second day<br>MTI-KS: Daily SMS about symptom awareness and knowledge of asthma (alternate days). Once SMS received, replied with True/False. Knowledge queries triggered another text that provided feedback on accuracy and fact about asthma	Outcomes:<br>• Lung Function - FEF 25–75% - MTI-KS vs. TAU Cohen's $d = 1.36$, $P = .036$.<br>• QoL -PAQLQ – MTI-KS vs. TAU; $P < .05$<br>• Engagement: 87% (75-99%)<br>• Attrition: MTI-KS 40% vs. MTI-S 60% vs. TAU 50%
Yun et al. (2013)	Study: RCT Study;<br>Platform: SMS<br>Duration: 3 months<br>Interactivity: SMS;<br>Recruited: Private paediatric asthma clinic	Participants *N*: 30 (10 each);<br>Demographics:10-16 years<br>Mean age: 13 years<br>Female: 57%<br>Asthma: undefined mid-severe persistent<br>Country: United States of America	TAU: No SMS<br>MTI-K: Text re symptom knowledge every second day<br>MTI-KS: Daily SMS about symptom awareness and knowledge of asthma (alternate days). Once SMS received, replied with True/False. Knowledge queries triggered another text that provided feedback on accuracy and fact about asthma	Outcomes:<br>• Lung Function - FEF 25–75% - MTI-KS vs. TAU - Cohen's $d = 0.85$, $P < 0.09$<br>• QoL - PAQLQ – MTI-KS vs. TAU 0.56±0.34 vs. .50), *P* ?<br>• Engagement: 85.6% (36-100%)<br>• Attrition: MTI-KS 30% vs. MTI-K 20% vs. TAU 40%

Abbreviations:

MTI: Mobile Technology Intervention
MTI-K: Mobile Technology Intervention – Knowledge
MTI-KS: Mobile Technology Intervention – Knowledge/Symptom
MTI-S: Mobile Technology Intervention - Symptom
PAQLQ: Pediatric Asthma Quality of Life Questionnaire
QoL: Quality of Life
RCT: Randomised Control Trial
SMS: Short Message Service
TAU: Treatment as Usual
