# Supplementary material for: Mobile Technology Interventions for Asthma Self-Management: Systematic Review and Meta-Analysis
Source: JMIR Mhealth Uhealth. 2017 May 2;5(5):e57. doi: 10.2196/mhealth.7168 (PMC5434254; doi:10.2196/mhealth.7168)
Supplement: Multimedia Appendix 4 [file mhealth_v5i5e57_app4.pdf]

Table A4.1 Study design characteristics of included studies

Lead Author	Year	RCT Design	Recruitment	Randomisation Method	Blinding
Ostojic	2005	2-Arm, Equal Size, Parallel	Single-site Pulmonary clinic	Random Number Computer Generation	Unknown
Liu	2007	2-Arm, Equal Size, Parallel	Single-site Outpatient clinic	Unknown	Unknown
Prabhakaran	2010	2-Arm, Equal Size, Parallel	Single-site Research Hospital	Drawn from envelope	Unknown
Strandbygaard	2010	2-Arm, Equal Size, Parallel	Advertisement in local paper	Block randomisation, block size 6	Unknown
Lv	2012	3-Arm, Equal Size, Parallel	Single-site Respiratory Research Hospital	Unknown	Unknown
Petrie	2012	2-Arm, Non-Equal Size, Parallel	Advertisement in Asthma Pamphlets and website	Random Number Computer Generation	Concealed to investigators until allocation
Ryan	2012	2-Arm, Non-Equal Size, Parallel	Multi-site (32) General Practise Clinics	Stratified by practice and centrally block randomised, block size of 2-4.	Concealed to investigators until allocation
Yun	2012	3-Arm, Equal Size, Parallel	Single-site Private Paediatric Asthma Clinic	Unknown	Unknown
Yun	2013	3-Arm, Equal Size, Parallel	Single-site Private paediatric asthma clinic	Unknown	Unknown
Cingi	2015	2-Arm, Equal Size, Parallel	Multi-site Pulmonary Research Hospital	Random Number Computer Generation	Patients blind to Intervention. Physicians blind to patient.

Zairina	2016	2-Arm, Equal Size, Parallel	Multi-site Antenatal Hospital Clinic	Stratified by asthma severity and block randomised, block sizes of 4-6.	Concealed to investigators until allocation
