# Supplementary material for: Mobile Technology Interventions for Asthma Self-Management: Systematic Review and Meta-Analysis
Source: JMIR Mhealth Uhealth. 2017 May 2;5(5):e57. doi: 10.2196/mhealth.7168 (PMC5434254; doi:10.2196/mhealth.7168)

Figure A5.1. Funnel plot of the standardised error in medication adherence between MTI and PB group

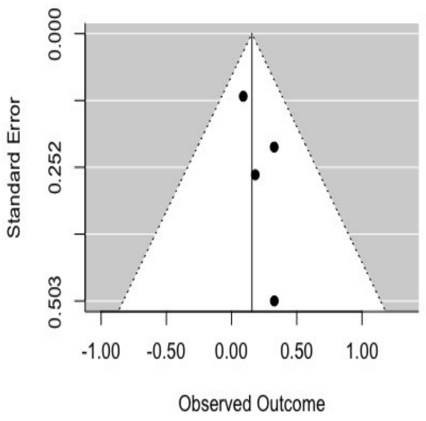

Figure A5.2. Funnel plot of the standardised error in adherence to symptom monitoring between MTI and PB group

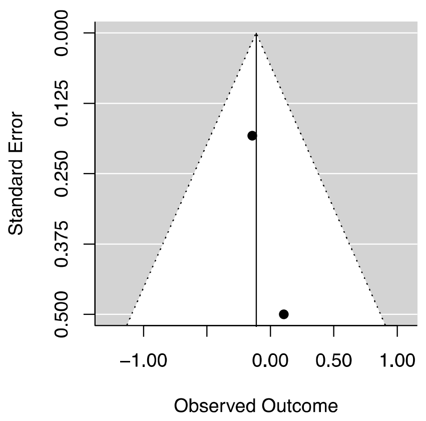

Figure A5.3. Funnel plot of the standardised error in lung function between MTI and PB group

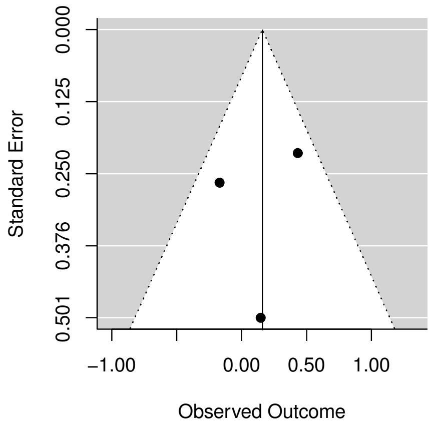

Figure A5.4. Funnel plot of the standardised error in quality of life between MTI and PB group

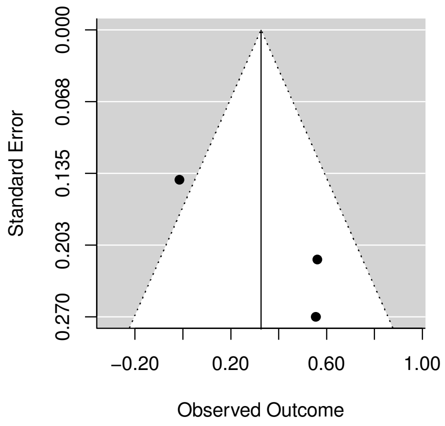

Figure A5.5. Funnel plot of the standardised error in asthma control between MTI and PB group

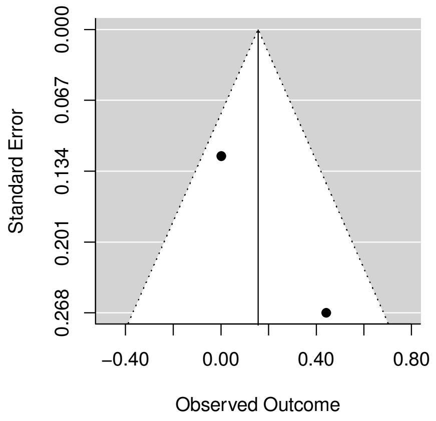

Figure A5.6. Funnel plot of the standardised error in unscheduled visits between MTI and PB group

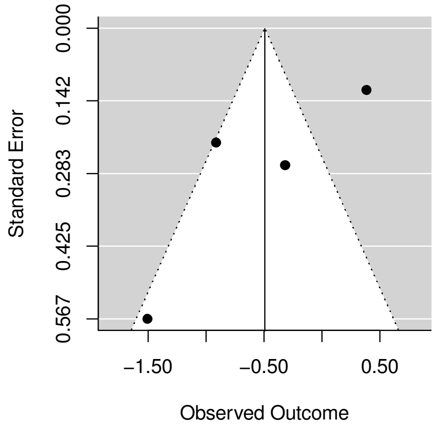

Figure A5.7. Funnel plot of the standardised error in attrition between MTI and PB group

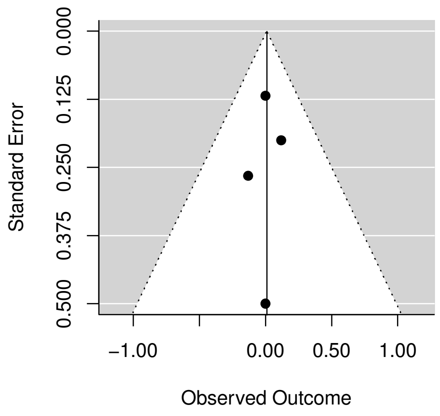

Figure A5.8. Funnel plot of the standardised error in medication adherence between MTI and TAU group

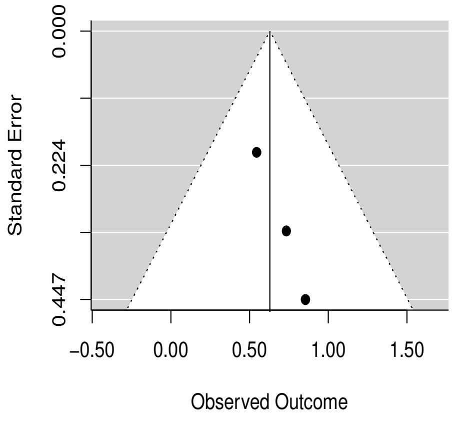

Figure A5.9. Funnel plot of the standardised error in lung function between MTI and TAU group

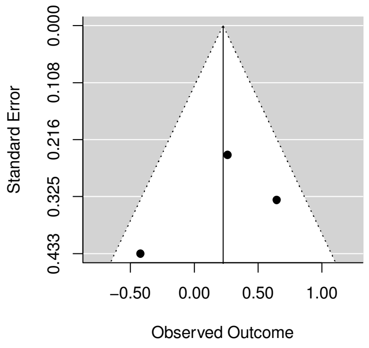

Figure A5.10. Funnel plot of the standardised error in quality of life between MTI and TAU group

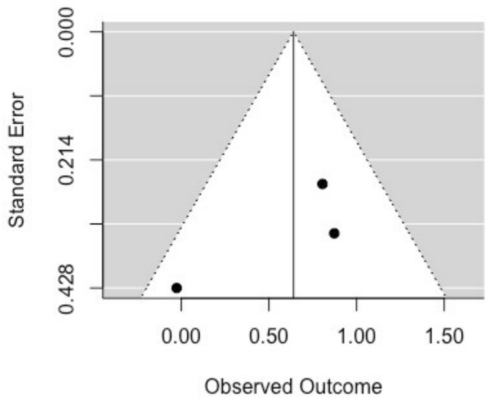

Figure A5.11. Funnel plot of the standardised error in asthma control between MTI and TAU group

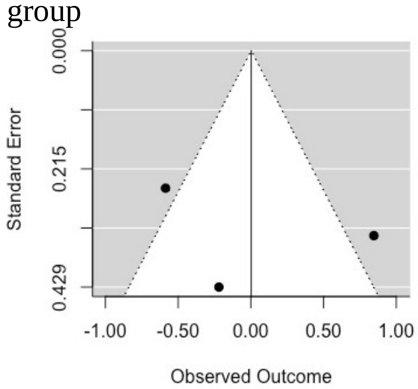

Figure A5.12. Funnel plot of the standardised error in well controlled asthma between MTI and TAU group

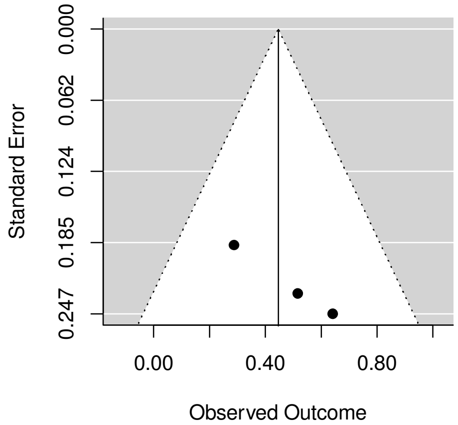

Figure A5.13. Funnel plot of the standardised error in unscheduled visits between MTI and TAU group

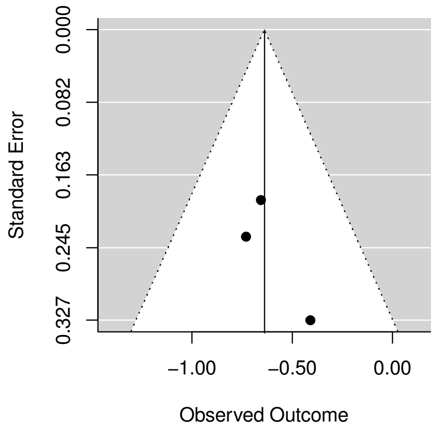

Figure A5.14. Funnel plot of the standardised error in attrition between MTI and TAU group

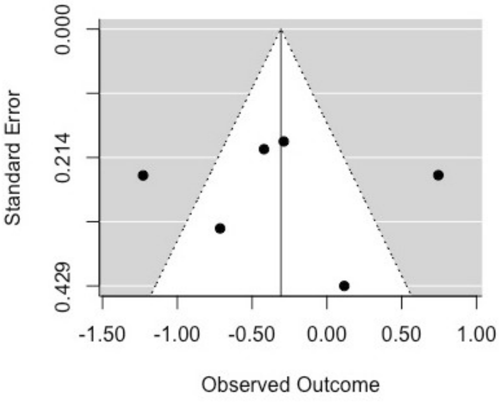

Supplement: Multimedia Appendix 5 [file mhealth_v5i5e57_app5.pdf]
