# Supplementary material for: Mobile Technology Interventions for Asthma Self-Management: Systematic Review and Meta-Analysis
Source: JMIR Mhealth Uhealth. 2017 May 2;5(5):e57. doi: 10.2196/mhealth.7168 (PMC5434254; doi:10.2196/mhealth.7168)

Table A6.1. Hedges' *g* and tests of heterogeneity of MTI for Attrition.

Attrition	k	N	Hedges' *g* (95%CI)	P^a	*Q*	P^b	I^2 (95% CI)
MTI vs							
Paper-based	4	457	0.01 (-0.17, 0.19)	.92	0.58	0.90	<.01 (<.01, 64.89)
Standard treatment	6	445	-0.31 (-0.86, 0.25)	0.28	35.66	<.001	86.61 (64.41, 97.77)

a: P-value of Hedges' *g* effect size
b: P-value of test for heterogeneity
k: number of studies
N: Total sample size across included studies
95%CI: 95% confidence intervals around the hedges' *g* effect size
Q: Measure of heterogeneity
I^2: measure of heterogeneity

Figure A6.1. Forest plot of the standardised mean difference in attrition between MTI and paper-based group.

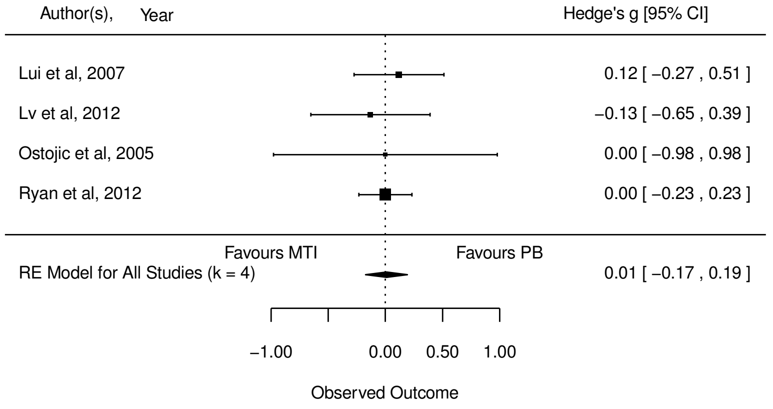

Figure A6.2. Forest plot of the standardised mean difference in attrition between MTI and standard treatment group.

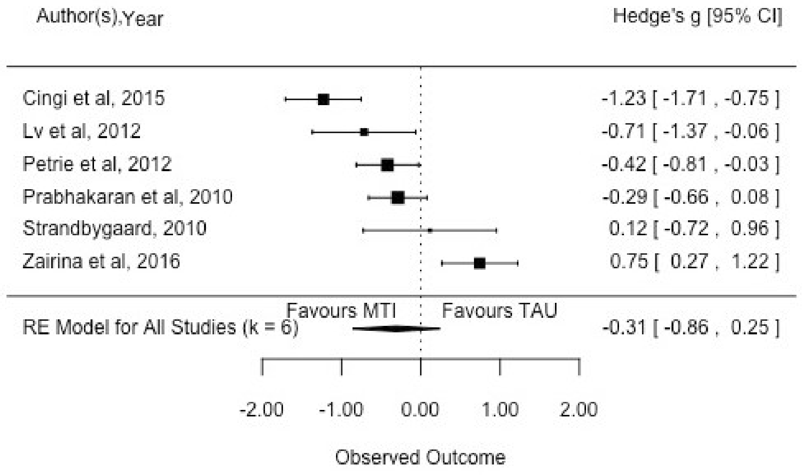

Supplement: Multimedia Appendix 6 [file mhealth_v5i5e57_app6.pdf]
